# Metabolic capacities of large “pillotinaceous” spirochetes from termite guts and their placement among ﻿*Breznakiellaceae*

**DOI:** 10.1186/s12915-026-02591-x

**Published:** 2026-04-07

**Authors:** Sebastian C. Treitli, Undine S. Mies, Renate Radek, Patricia A. Zinnhardt, Joana Maria Kästle Silva, Lisa Reuter, Natalie A. Röhr, Katja Platt, Vincent Hervé, Rudy Plarre, Mario Marini, Andreas Brune

**Affiliations:** 1 Research Group Insect Gut Microbiology and Symbiosis, Max Planck Institute for Terrestrial Microbiology, Marburg, Germany; 2 Institute of Biology/Zoology, Free University of Berlin, Berlin, Germany; 3 Université Paris-Saclay, INRAE, AgroParisTech, UMR SayFood, Palaiseau, 91120 France; 4 Federal Institute for Materials Research and Testing (BAM), Berlin, Germany; 5 Department of Biological, Geological and Environmental Sciences, University of Bologna, Bologna, Italy

**Keywords:** Spirochetes, Phylogenomics, Single-cell techniques, *Pillotinaceae*, Gut microbiota, Ultrastructure

## Abstract

**Background:**

Spirochetes are the most abundant bacterial group in the hindgut of termites. The largest species, with cell lengths of up to 100 µm, have been provisionally classified in the family “*Pillotinaceae*” based exclusively on morphological traits. However, in the absence of cultured representatives, their phylogenetic position and metabolism remain entirely unknown.

**Results:**

We investigated phylogeny and metabolic capacities of “pillotinaceous” spirochetes using single-cell techniques, electron microscopy, and fluorescence in situ hybridization. All sequences of large spirochetes obtained from various termites fell into four distinct, well-supported clusters within the family *Breznakiellaceae*. Based on ultrastructural features, three of the clusters were assigned to the genera *Pillotina*, *Hollandina*, and the newly established genus *Hollandinoides;* a fourth cluster was tentatively assigned to the genus *Clevelandina*. Functional analysis of the single-cell genomes of *Pillotina corrugata* sp. nov., *Hollandina grandis* sp. nov., and *Hollandinoides gharagozlouae* gen. nov. sp. nov., combined with comparative genomics of other uncultured relatives, demonstrated differences in the capacity to degrade cellulose, hemicelluloses, and dextrins. While members of the genus *Pillotina* have a fermentative metabolism, members of the other genera encode a Wood–Ljungdahl pathway and, in the case of *Hollandina*, a group-III nitrogenase, suggesting roles in reductive acetogenesis and nitrogen fixation.

**Conclusions:**

Our results provide the first molecular data on pillotinaceous spirochetes. We show that the three genera covered in our study belong to the family *Breznakiellaceae*, which harbors the majority of termite-gut spirochetes. Comparative genome analysis indicated that the large spirochetes in termite guts have distinct roles in symbiotic digestion.

**Supplementary Information:**

The online version contains supplementary material available at 10.1186/s12915-026-02591-x.

## Background

The hindgut of termites has the highest abundance and diversity of spirochetes of any microbial habitat. Individual lineages of termite gut spirochetes are either free-swimming in the hindgut fluid or associated with flagellated protists [1–3]. Spirochetes can make up more than half of the prokaryotic gut microbiota in certain wood-feeding termite species and are considered important players in the symbiotic digestion of lignocellulose [4–6]. In live mounts of hindgut suspensions, spirochetal cells are easily recognized by their conspicuous helical shape and high motility. The more abundant morphotypes are small, but most termite species also harbor fewer but extremely large forms with a cell diameter of up to 1.5 µm and a length greater than 100 µm [7–9].

Despite their conspicuous morphology and many unusual ultrastructural features, individual species of the phylum *Spirochaetota *can only be identified by molecular techniques. The earliest 16S rRNA gene-based studies of termite-gut spirochetes revealed two distinct phylogenetic clades in the radiation of the genus *Treponema*, which were referred to as “termite cluster I” and “termite cluster II” [10, 11] and recently reclassified in the families *Breznakiellaceae *and *Treponemataceae *(order *Treponematales*), respectively [12]. The majority of termite-gut spirochetes belong to the family *Breznakiellaceae*, where they form numerous host-specific lineages. The representatives of *Treponemataceae *form only a small clade that comprises intracellular symbionts of termite-gut flagellates lacking the typical spirochetal morphology [13, 14]*.* In addition, several other lineages of termite-gut spirochetes, comprising members of *Sphaerochaetaceae*, *Alkalispirochaetaceae*, and uncultured *Leptospirales*, have been detected in cultivation-based and metagenomic studies [5, 15–17]; they fall outside the *Treponematales *and have so far received little attention.

Already in the late nineteenth century, Joseph Leidy observed large free-swimming spirochetes in termite guts [18, 19]. Their ultrastructural features were first documented by Hollande and Gharagozlou, who described *Pillotina calotermitidis* [8] and *Diplocalyx calotermitidis* [20] from dry-wood termites (Kalotermitidae). Their observations were expanded by formal descriptions of these and additional species, including *Hollandina pterotermitidis* from *Pterotermes occidentis* and *Clevelandina reticulitermitidis* from subterranean termites (Rhinotermitidae) [7, 21]. Based on the unique morphological characteristics of these genera, the creation of a separate family, “*Pillotinaceae*”, has been proposed [22].

Like other termite gut spirochetes, also the large “pillotinaceous” forms possess periplasmic flagella that are inserted subterminally at each cell pole and wrap around the protoplasmic cylinder. These “endoflagella” are responsible for the helical shape and the high motility in viscous media typical for most members of the phylum *Spirochaetota *[23, 24]. However, pillotinaceous spirochetes differ from the smaller forms in several features, including a larger size and increased degree of multiplication of the periplasmic flagella in all species, and a combination of other morphological traits, such as a crenulated outer sheath, a deep invagination of the outer membrane (groove or “sillon”), and various decorations of cytoplasmic and outer membranes, which are used to differentiate between individual genera [7, 8, 20, 22].

The large spirochetes in termite guts are easily recognizable under both light and electron microscopy, yet not a single representative has been cultivated. Despite the absence of type strains, the species names of the above-mentioned morphotypes are considered validly published under the rules of the *International Code of Nomenclature for Prokaryotes* (ICNP). However, without molecular data, the relationship between the different genera and the validity of the family “*Pillotinaceae*” cannot be tested, and none of the species can be linked to the many genomes of uncultured termite-gut spirochetes that have been obtained in metagenomic studies and single-cell analyses of termite-gut microbiota (e.g., [5, 15, 25, 26]). Therefore, their phylogenetic position and metabolic capacities have remained entirely obscure.

In this work, we identified the large spirochetes in the hindgut of several lower termites using micromanipulation, single-cell genomics, and fluorescence in situ hybridization (FISH). We conducted phylogenetic and phylogenomic analyses and characterized the metabolic potential of selected representatives by functional genome analysis and comparative genomics. Based on the genome sequences, we describe a new genus and three new species under the rules of the *Code of Nomenclature of Prokaryotes Described from Sequence Data* (SeqCode) [27] and provide emended descriptions of existing genera and their taxonomic classification.

## Results

### Identification and phylogenetic position of large spirochetes

Using micromanipulation, we isolated large spirochetal cells from the gut contents of *Kalotermes flavicollis* (8 individual cells)*, Kalotermes italicus* (5 individual cells)*, Incisitermes tabogae* (3 individual cells)*, Incisitermes* aff. *schwarzi* (5 individual cells), and *Reticulitermes flavipes* (9 individual cells). The DNA of each cell was amplified using whole genome amplification (WGA), and the 16S rRNA genes were sequenced with universal *Bacteria* primers. The resulting single-cell amplicon (SCA) sequences were placed into a reference alignment of 16S rRNA genes of *Spirochaetota* that comprised representatives from a wide range of termites and other arthropods.

**Fig. 1:**
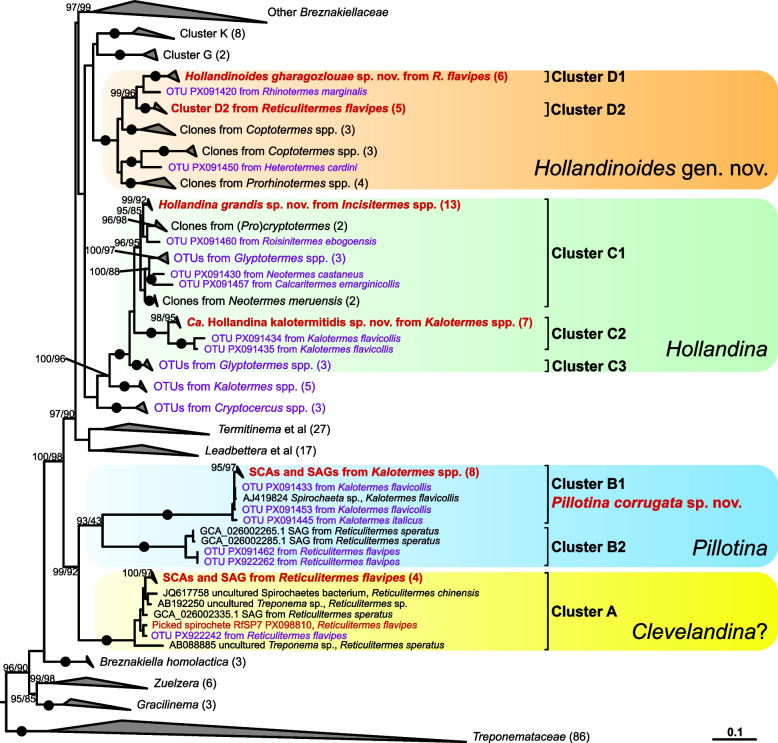
Maximum-likelihood (ML) phylogenetic tree showing the relationship of our isolated bacterial 16S rRNA phylotypes within the family *Breznakiellaceae*. Values at the nodes represent ML ultrafast bootstrap support values (> 90%) and SH-aLRT scores (> 80). Bullets represent full node support (ML: 100%; SH-aLRT: 100%). Sequences obtained from single cells are in red and sequences from investigated amplicon libraries are in purple. The number next to the collapsed clade represents the number of sequences in that clade. The tree was rooted with other members of *Treponematales*. Scale bar, 0.1 expected substitutions per site. A fully expanded version of the tree can be found in Additional File 1: Fig. S1

Phylogenetic analysis revealed that all SCAs of large spirochetes fall into the radiation of the family *Breznakiellaceae*, where they form four well-supported clusters (clusters A–D; Fig. 1, Additional File 1: Fig. S1). Cluster A consists exclusively of sequences from the genus *Reticulitermes*, including three SCAs and one single-cell amplified genome (SAG) from *R. flavipes*. Cluster B comprises two subclades, one with SCAs and SAGs from the genus *Kalotermes* (*Pillotina corrugata* sp. nov.; cluster B1), the other with representatives from the genus *Reticulitermes* (cluster B2), including two SAGs previously recovered from *R. speratus* [25]. Cluster C contains sequences from numerous members of the family Kalotermitidae (cluster C1), including SCAs and SAGs from the genus *Incisitermes* (*Hollandina grandis* sp. nov.), and a subclade (cluster C2) with SCAs from the genus *Kalotermes* (*Candidatus* Hollandina kalotermitidis). Cluster D consists exclusively of sequences from Rhinotermitidae, including a lineage comprising SCAs and a SAG from *R. flavipes* (*Hollandinoides gharagozlouae* gen. nov. sp. nov.; cluster D1) and one containing only SCAs from *R. flavipes* (cluster D2).

### Linking phylogeny to morphology

**Fig. 2:**
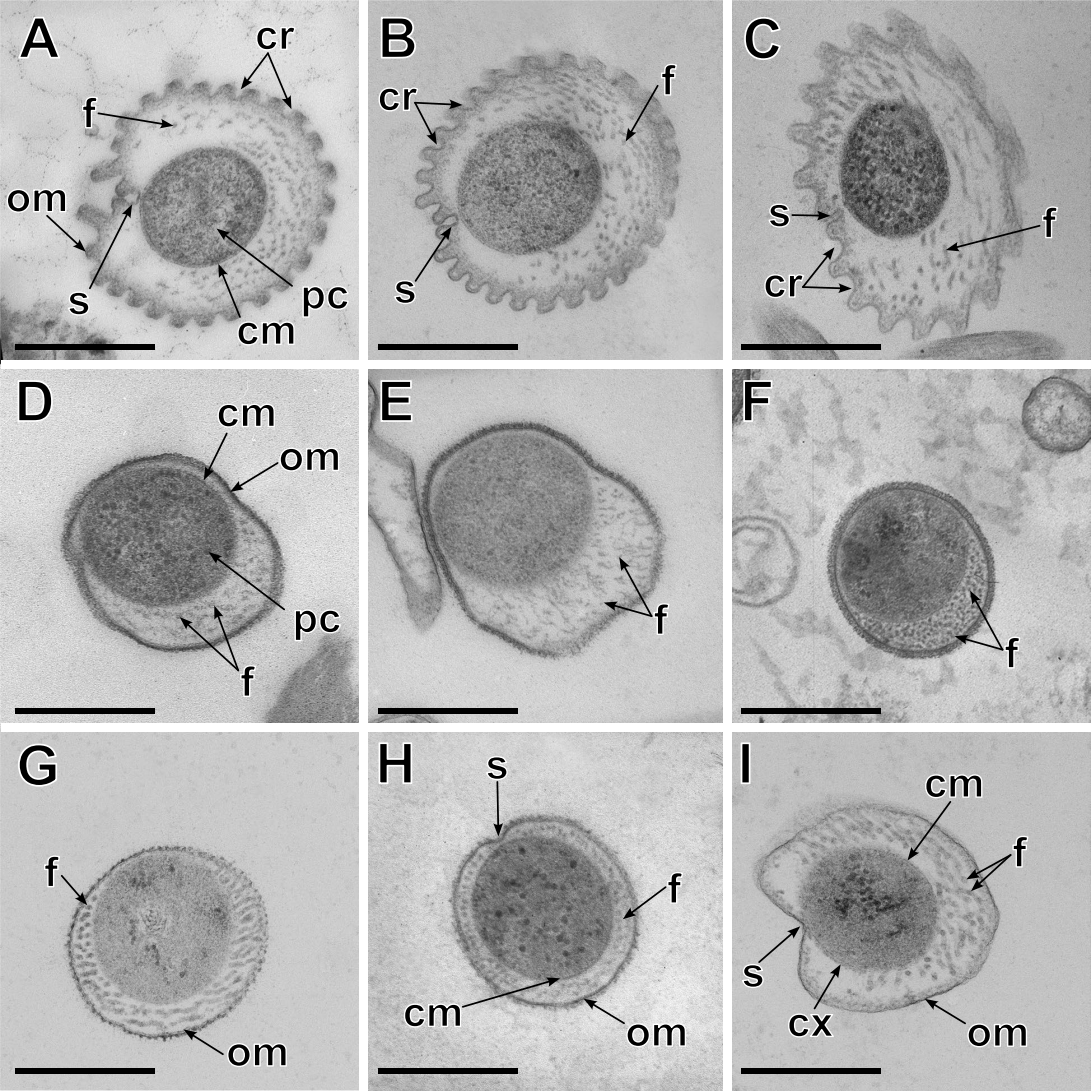
Transmission electron micrographs of spirochete morphotypes identified in different termites. **A** *Pillotina* from *Kalotermes flavicollis,* showing the protoplasmic cylinder (pc), numerous flagella (f), outer membrane (om), crenulations on the outer membrane (cr), cytoplasmic membrane (cm), and sillon (s). **B** *Pillotina* from *Kalotermes italicus.* **C** *Pillotina* from *Reticulitermes flavipes.* **D** *Hollandina* from *K. flavicollis.* **E** *Hollandina* from *K. italicus.* **F** *Hollandina* from *Incisitermes tabogae.* **G** *Hollandina*-like morphotype from *Reticulitermes flavipes.* **H** *Hollandina*-like morphotype with a sillon from *Reticulitermes flavipes.* **I** *Clevelandina*-like morphotype with a sillon and a calyx (cx) from *Reticulitermes flavipes*

Electron microscopy (EM) of hindgut suspensions revealed that the investigated termites harbor four morphotypes of large spirochetes (Fig. 2). All morphotypes have numerous periplasmic flagella but differ in the structure of the outer membrane and additional features. One morphotype, found in *K. flavicollis*, *K. italicus*, and *R. flavipes* (Fig. 2A–C), has a strongly crenulated outer membrane with an underlying electron-dense layer, which is attached to the cytoplasmic membrane at one point, creating an axial groove (sillon) along the cell body. This combination of traits is consistent with the descriptions of *Pillotina calotermitidis* from *Postelectrotermes praecox* and similar morphotypes observed in both *Kalotermes* and *Reticulitermes* spp. [8, 28]. A second morphotype, present in *K. flavicollis*, *K. italicus*, *I. tabogae,* and *R. flavipes* (Fig. 2D–G), lacks a sillon and has a smooth outer membrane decorated with a thick outer coat. These characteristics are consistent with the descriptions of *Hollandina pterotermitidis* from *Pterotermes occidentis* and similar morphotypes observed in other kalotermitids [7, 21, 29]. The two remaining morphotypes were found exclusively in *R. flavipes*. One resembles the morphotype of *Hollandina* but possesses a discrete sillon (Fig. 2H). The other has a smooth outer membrane with a thick inner coat that forms a sillon, and a cup-like structure (calyx) partially engulfing the protoplast (Fig. 2I). The same morphotype has been previously observed in *R. flavipes* [30] and matches the description of *Clevelandina reticulitermitidis* from *Reticulitermes tibialis* [7]*.*

**Fig. 3:**
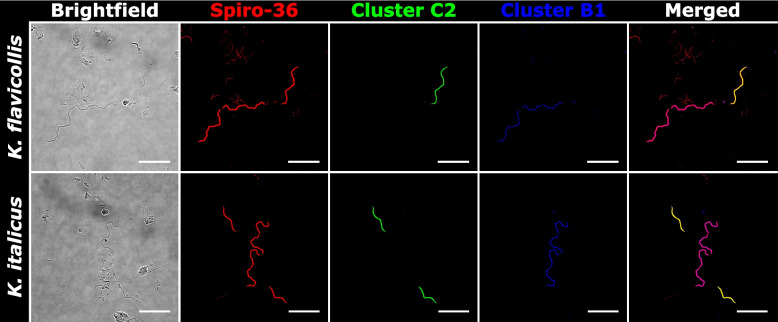
FISH of spirochetes in the gut contents of *Kalotermes* spp. using different oligonucleotide probes. The Spiro-36 probe targets the majority of termite gut spirochetes. Phylotypes from *K. flavicollis* and *K. italicus* belonging to cluster C2 are targeted by the Kf-Hol-193 probe*.* Phylotypes from *K. flavicollis* and *K. italicus* belonging to cluster B1 are targeted by the Kf-Pil-1449 probe*.* Probe sequences are given in Table 1. All scale bars are 20 µm

**Fig. 4:**
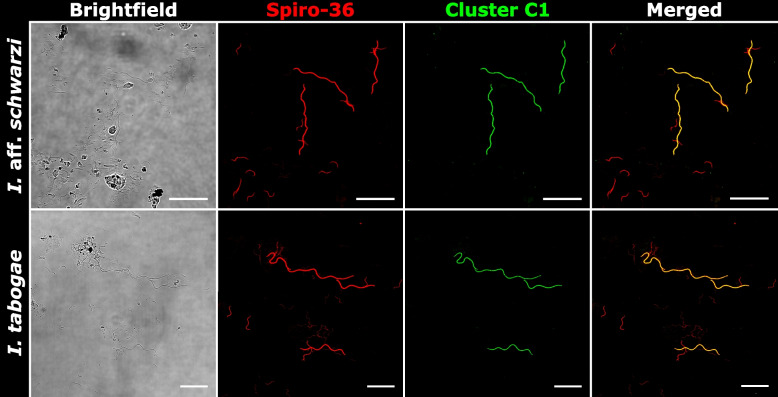
FISH of spirochetes in the gut contents of *Incisitermes* spp. using different oligonucleotide probes. The Spiro-36 probe targets the majority of termite gut spirochetes. Phylotypes from *I. tabogae and I. aff. schwarzi* belonging to cluster C1 are targeted by the It-Hol-183 probe*.* Probe sequences are given in Table 1. All scale bars are 20 µm

To link the 16S rRNA gene sequences obtained by single-cell analysis to their corresponding morphotypes and to determine whether all phylotypes of large spirochetes were detected, we conducted fluorescence in situ hybridization (FISH) with a previously published general probe [31] that targets the majority of termite gut spirochetes, except the representatives of cluster D, and several specifically designed oligonucleotide probes against individual phylogenetic clusters (Table 1). In the case of *K. flavicollis* and *K. italicus*, two specific probes targeting the phylotypes of *Pillotina* (subcluster B1) and *Hollandina* (subcluster C2) obtained from these termites allowed us to differentiate the two distinct morphotypes of large spirochetal cells present in the respective samples (Fig. 3). None of the large spirochetal cells hybridized exclusively with the general probe, suggesting that the *Kalotermes* spp. investigated in our study are colonized only by members of the genera *Pillotina* and *Hollandina*, which is consistent with the ultrastructural data. In the case of *I. tabogae* and *I.* aff. *schwarzi*, a specific probe targeting the phylotype of *Hollandina* (subcluster C1) obtained from these termites identified a single morphotype of large spirochetes in the respective samples (Fig. 4). Again, the congruence of the signal with that of the general probe confirmed that additional phylotypes of large spirochetes are absent. All samples contained smaller spirochetal cells that hybridized exclusively with the general probe.

**Table 1:** Oligonucleotide probes used for the fluorescence in-situ hybridization of large spirochetes in different termites, the optimal formamide concentrations, and the (sub)cluster and phylotypes targeted by the respective probe

Name	Probe sequence (5'−3')	Optimal formamide concentration	(Sub-) cluster	Targeted phylotypes
It-Hol-183	CCATGCCACAGCACGATAAG	40%	C1	*Hollandina grandis* from *Incisitermes* spp.
Kf-Hol-193	GAGCCACAGCCCCTTTCCT	30%	C2	*Hollandina* from *Kalotermes* spp.
Kf-Pil-1449	GCAGCGCCCTCCTTTTACAA	30%	B1	*Pillotina corrugata* from *Kalotermes* spp.
Rf-Clev-140	CTAACAGATATCCCCAACCC	30%	A	Putative *Clevelandina* from *Reticulitermes* spp.
Rf-Pil-129	CCCAACCTCTCGGGTAGATT	30%	B2	*Pillotina* from *Reticulitermes* spp.
Rf-HolD-1041	CCATGCAGCACCTGTAGT	30%	D	*Hollandinoides* from *Reticulitermes flavipes*
Rf-HolD1-990	GGCTTCCCCACTATGTCAAA	20%	D1	*Hollandinoides gharagozlouae* from* Reticulitermes flavipes*

**Fig. 5:**
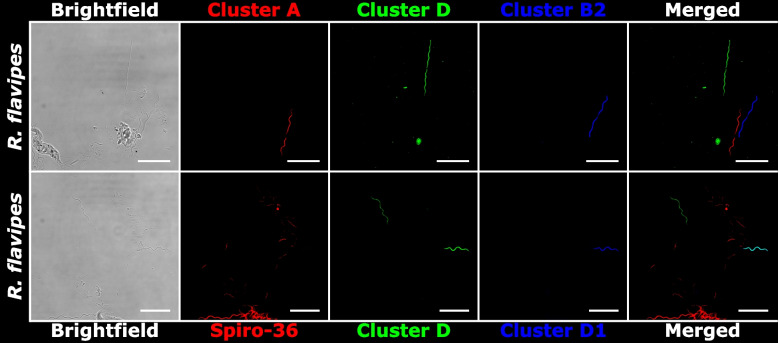
FISH of spirochetes in the gut contents of *Reticulitermes flavipes* using different oligonucleotide probes. Probe Spiro-36 targets the majority of termite gut spirochetes, with the exception of representatives from cluster D. Phylotypes from *R. flavipes* belonging to the cluster A are targeted by the Rf-Clev-140 probe. Phylotypes from *R. flavipes* belonging to the cluster D are targeted by the Rf-HolD-1041 probe. Phylotypes from *R. flavipes* belonging to the cluster B2 are targeted by the Rf-Pil-129 probe*.* Phylotypes from *R. flavipes* belonging to the cluster D1 are targeted by the Rf-HolD1-990. Probe sequences are given in Table 1. All scale bars are 20 µm

In the case of *R. flavipes*, a set of specific probes targeting the phylotypes in clusters A, B2, and D that are represented in this termite species clearly differentiated three morphotypes of large spirochetes (Fig. 5 top row). Notably, the morphotype targeted by the probe against subcluster B2, represented by only a few amplicon sequences from *R. flavipes*, was very rare, which would explain the absence of this phylotype from the SCAs obtained from this termite and the low abundance of cells with the morphology of *Pillotina* in ultrathin sections of *R. flavipes* (Fig. 2C). A combination of the probe for cluster D with an additional probe (Rf-HolD1-990) against subcluster D1 allowed us to distinguish two morphotypes that represent subclusters D1 and D2, both of which did not hybridize with the general probe (Fig. 5, bottom row).

### Genome sequencing, assembly, and phylogenomic analysis

Two WGA products of *Pillotina corrugata* from *K. flavicollis,* two WGA products of *Hollandina grandis* from *I. tabogae,* and two WGA products from *R. flavipes* were selected for short-read genome sequencing using an Illumina platform. After genome assembly and decontamination, we obtained a SAG for *Pillotina corrugata* with an assembly size of 3.06 Mbp and a GC content of 43.2 mol%. Based on CheckM, the genome completeness was estimated at 97.4% with no contamination. In the case of *Hollandina grandis*, one of the two WGA products (ItSP3) was contaminated with other bacteria and was discarded. After assembly and decontamination, the remaining sample yielded a SAG with an assembly size of 3.55 Mbp and an average GC content of 49.1 mol%. The genome completeness was estimated at 95.5% with 1.7% contamination. Sample RfSP5 from *R. flavipes* yielded a SAG for *Hollandinoides gharagozlouae* with an assembly size of 3.69 Mbp and a GC content of 47 mol%. Based on CheckM, the genome completeness was estimated at 91.7% with 0.1% contamination. The second WGA product from *R. flavipes*, RfSP9, yielded a SAG with an assembly size of 3.45 Mbp, an average GC content of 48.7 mol%, and a genome completeness of 78.3% with 3.4% contamination (Table 2).

**Table 2:** General features of the single-cell assembled genomes obtained in this study. Completeness and contamination were estimated using CheckM (CheckM2 in parentheses). Estimated genome size is based on assembly size and completeness

SAG	Scaffolds	Assembly size (bp)	Estimated genome size (Mbp)	N50 (kbp)	GC content (mol%)	Completeness (%)	Contamination (%)
*Pillotina corrugata* KfSPG140	147	3,056,337	3.14	49.0	43.2	97.4 (94.3)	0.0 (0.2)
*Hollandina grandis* ItSP2	312	3,553,180	3.72	21.6	49.1	95.5 (84.7)	1.7 (1.3)
*Hollandinoides gharagozlouae* RfSP5	121	3,691,541	4.02	42.6	47	91.7 (92.9)	0.1 (0.1)
RfSP9 from *Reticulitermes flavipes*	211	3,452,027	4.41	23	48.7	78.3 (78.8)	3.4 (1.5)

**Fig. 6:**
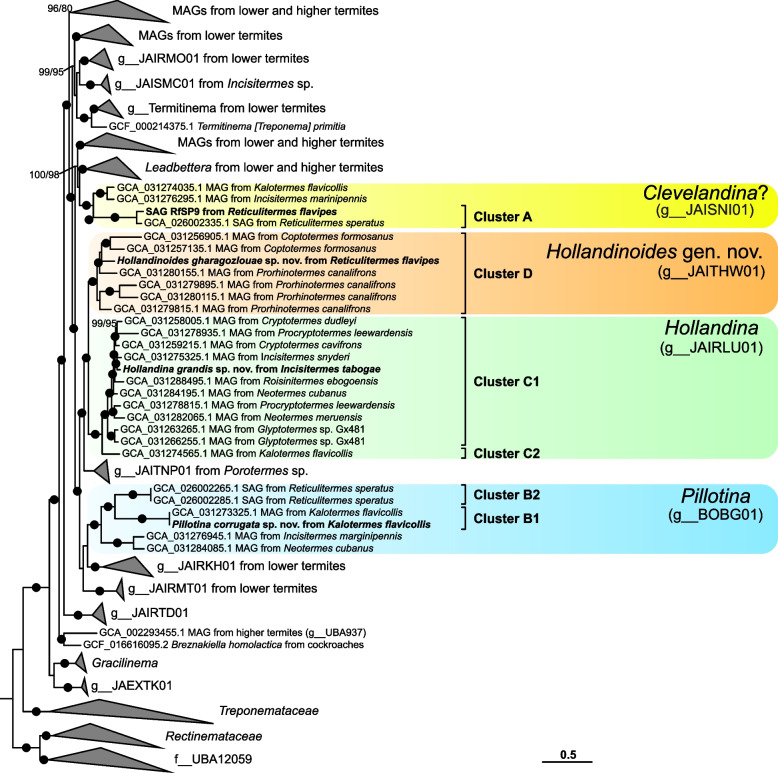
Maximum-likelihood (ML) phylogenomic tree showing the relationship of *Pillotina corrugata, Hollandina grandis*, and *Hollandinoides gharagozlouae* to other representatives of the family *Breznakiellaceae*. Values at the nodes represent ML ultrafast bootstrap support values (> 90%) and SH-aLRT scores (> 80). Bullets represent full node support (ML: 100%; SH-aLRT: 100%). The newly obtained genome sequences are in bold. The tree was rooted with other representatives of *Spirochetales* (not shown)*.* Scale bar, 0.5 expected substitutions per site

In all cases, the 16S rRNA genes in the SAGs were identical to the SCA sequences obtained from the same samples. Phylogenomic analysis confirmed that all SAGs represent species that fall into the family *Breznakiellaceae *(Fig. 6). The genome of *Pillotina corrugata* is almost identical (99.7% average nucleotide identity) to a MAG (GCA_031273325.1) previously obtained from the gut content of *K. flavicollis* [15], and falls into a genus-level clade (g__BOBG01) that also comprises MAGs from other Kalotermitidae (*Incisitermes marginipennis* and *Neotermes cubanus*) and two SAGs from *R. speratus. The* genome of *Hollandina grandis* falls into another genus-level clade (g__JAIRLU01) of MAGs obtained from a wide range of Kalotermididae. A sister clade (g__JAITHW01) harbors the SAG of *Hollandinoides gharagozlouae* from *R. flavipes* and several MAGs obtained from other Rhinotermitidae. SAG RfSP9 falls into a genus-level clade (g__JAISNI01) comprising a SAG from *R. flavipes* and two MAGs from Kalotermitidae. Because of the low genome completeness, we did not attempt a full metabolic reconstruction of this MAG but we included it in a comparative genome analysis of important features. The topology and composition of the *Pillotina, Hollandina*, and *Hollandinoides* clades match the corresponding clusters in the 16S rRNA gene tree (Fig. 1).

### Metabolic capacities of the new species

**Fig. 7:**
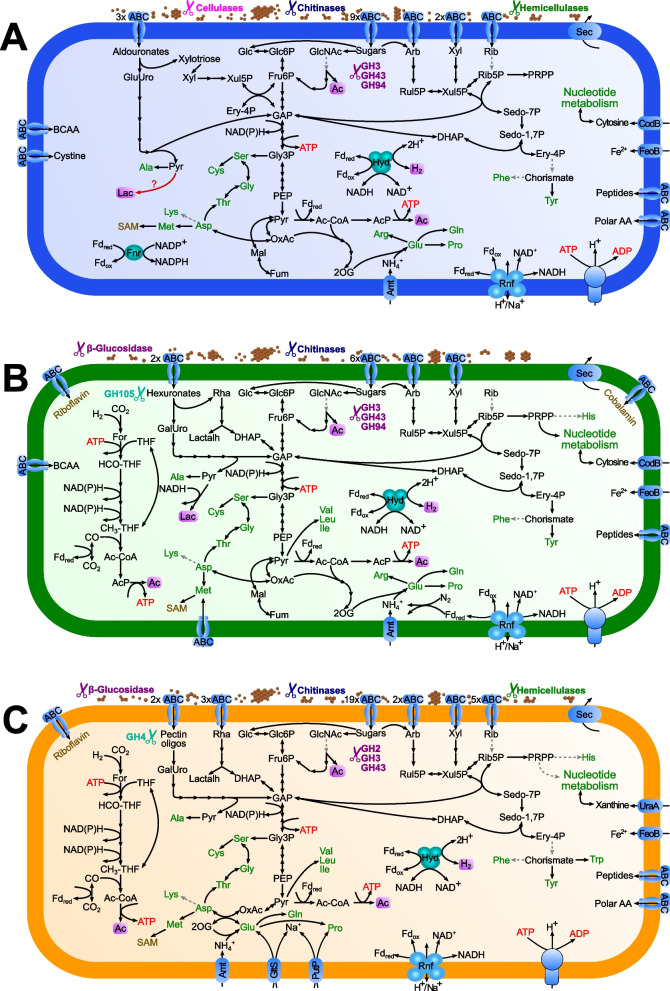
Predicted metabolic pathways of the new species. **A** *Pillotina corrugata*, **B** *Hollandina grandis.* **C** *Hollandinoides gharagozlouae*. Amino acids are shown in green; vitamins and cofactors are shown in pink. Pathways missing some enzymes are marked with a gray dashed arrow. Non-standard abbreviations: Ac, acetate; Ac-CoA, acetyl-coenzyme A; AcP, acetyl phosphate; Arb, arabinose; BCAA, branched-chain amino acid; DHAP, dihydroxyacetone phosphate; Ery-4P, erythrose 4-phosphate; For, formate; Fnr, ferredoxin:NADP + oxidoreductase; Fru6P, fructose 6-phosphate; Fum, fumarate; GAP, glyceraldehyde 3-phosphate; GalUro, galacturonate; Glc, glucose; Glc6P, glucose 6-phosphate; GlcNAc, *N*-acetyl glucosamine; GluUro, glucouronate; Gly3P, glycerate 3-phosphate; Lac, lactate; Lactalh, lactaldehyde; Mal, malate; OxAc, oxaloacetate; 2OG, 2-oxoglutarate; PEP, phosphoenolpyruvate; Pyr, pyruvate; Rha, rhamnose; Rib, ribose; Rib5P, ribose 5-phosphate; Rnf, ferredoxin:NAD^+^ oxidoreductase complex; Rul5P, ribulose 5-phosphate; Sedo-7P, sedoheptulose 7-phosphate; Sedo-1,7P, sedoheptulose 1,7-diphosphate; THF, tetrahydrofolate; Xyl, xylose; Xyl5P, xylose 5-phosphate. End products of the energy metabolism are highlighted

The genomes of *P. corrugata* and *Hollandinoides gharagozlouae* both encode a high number of glycosyl hydrolases (GH) (41 GHs from 27 GH families and 42 GHs from 27 GH families, respectively), whereas their number in the genome of *H. grandis* is much lower (23 GHs from 17 families; Additional File 2: Table S1, S2 and S3). Homologs with a secretion signal present in *P. corrugata* comprise an endoglucanase (GH5), an endoxylanase (GH10), a chitinase (GH18), an endo-mannosidase (GH26), a β-galactosidase (GH39), and an α-glucuronidase (GH67). In *Hollandinoides gharagozlouae*, the number of secreted GHs was slightly higher, including two β-glucosidases (GH3), an endoxylanase (GH10), two chitinases (GH18) an endo-mannosidase (GH26), and three endo-xylanases (GH30), whereas *H. grandis* possesses only a single β-glucosidase (GH3), a chitinase (GH5_48), and a lichenase/laminarinase (GH16_3). A homolog of GH73 involved in chitin degradation is present in all species (Fig. 7). While all genomes encode a large number of ATP-binding cassette (ABC)-type sugar transporters, their number is about double as high in *Hollandinoides gharagozlouae* (34) than in the other species (16 in *P. corrugata* and 10 in *H. grandis*). We confidently identified ABC transporters of xylose, arabinose, and aldouronates/hexuronates in all genomes, transporters of ribose in *P. corrugata* and *Hollandinoides gharagozlouae*, and several importers for pectin oligosaccharides and rhamnose in the genome of *Hollandinoides gharagozlouae* (Additional File 2: Table S4, S5 and S6). The substrate specificity of the remaining sugar transporters is unclear. All organisms encode putatively cytoplasmic β-glucosidases from various GH families that are involved in the degradation of sugar oligomers. One of the aldouronate transporters of *H. grandis* is part of an operon that contains a homolog of *yesR*, which is crucial for rhamnogalacturonan degradation in *Bacillus subtilis* [32], suggesting that *H. grandis* imports rhamnogalacturonan and possibly other hexuronates that are subsequently hydrolyzed by GH105.

All organisms convert the imported sugars to pyruvate via glycolysis (Fig. 7). Pentoses and aldouronates are shuttled into the glycolytic pathway via the pentose-phosphate pathway and the KDPG pathway. In *H. grandis and P. corrugata,* pyruvate is converted to acetyl-CoA by pyruvate:ferredoxin oxidoreductase (PFOR), and the production of acetate by phosphate acetyltransferase and acetate kinase provides additional ATP by substrate-level phosphorylation*.* In *Hollandinoides gharagozlouae*, which lacks phosphate acetyltransferase, acetate production and substrate-level phosphorylation may proceed via an ADP-forming acetyl-CoA synthetase (acdAB, Additional File 2: Table S6). All species possess an electron-confurcating hydrogenase of group A3, which catalyzes the concomitant reoxidation of NADH and reduced ferredoxin generated during the conversion of sugars to acetate and CO_2_. In addition, both *H. grandis* and *Hollandinoides gharagozlouae* possess all enzymes for the reduction of CO_2_ to acetyl-CoA via the Wood–Ljungdahl pathway (WLP), including a hydrogen-dependent CO_2_ reductase (HDCR) and a CO dehydrogenase-acetyl-CoA synthase (ACS) complex, which allows regeneration of NADH and reduced ferredoxin by reductive acetogenesis. A Na^+^-translocating ferredoxin:NAD + oxidoreductase (Rnf) complex and an F-type ATP synthase allow both species to generate additional ATP by electron-transport phosphorylation. The complex is present also in *P. corrugata*, where it may serve to adjust the redox balance between NADH and ferredoxin and to generate membrane potential. Notably, a ferredoxin-NADP + reductase (FNR) is present only in the genome of *P. corrugata.* A classical (NADH-dependent) lactate dehydrogenase (Ldh) is present only in *H. grandis*; in *P. corrugata*, we identified an Ldh family oxidoreductase with unclear substrate specificity.

The genomes of all species encode the pathways for de novo biosynthesis of 11 proteinogenic amino acids. Branched-chain amino acids can be synthesized only by *H. grandis* and *Hollandinoides gharagozlouae*, and all species lack the capacity to synthesize asparagine, histidine and phenylalanine, indicating a dependence on an external supply of amino acids as growth factors. All genomes contain an ammonium transporter (Amt), but only *H. grandis* possesses a complete operon (*nifADHKENB*) encoding a group-III nitrogenase, indicating the capacity to fix dinitrogen. While *nif-gene* homologues were completely absent in *Hollandinoides gharagozlouae*, *P. corrugata* possesses several homologs of group-IV nitrogenase genes (*nifB*, *nifD*, *nifK,* and *nifH*) that are not part of a gene cluster but spread across distant loci. While *nifB* and *nifH* are associated with an AAA-family ATPase and a radical-SAM protein, *nifK* and *nifD* are part of an operon encoding leucine-rich repeat proteins and a cysteine synthase that might be involved in sulfur metabolism [33, 34].

## Discussion

In the present study, we combined genomic data, fluorescence in situ hybridization, and ultrastructural characterization to provide a molecular basis for the taxonomic classification of the so-far-elusive pillotinaceous spirochetes in termite guts. We demonstrated that the large spirochetes colonizing the termite genera *Kalotermes*, *Incisitermes*, and *Reticulitermes* represent four distinct, genus-level lineages of *Breznakiellaceae*, a recently described family of *Treponematales *that harbors the majority of all termite gut spirochetes [12]. Comparative analysis of the high-quality genomes of *P. corrugata, H. grandis,* and *Hollandinoides gharagozlouae* revealed that the large spirochetes in termite guts differ in their metabolic potential, indicating distinct roles in symbiotic digestion.

### The genus *Pillotina*

The description of the type species, *Pillotina calotermitidis*, is based on a morphotype that occurs in the gut of *Postelectrotermes* [syn. *Calotermes, Kalotermes*] *praecox* [7, 8]. Large spirochetes with a similar morphology have also been observed in *R. flavipes*, *R. hesperus,* and *Incisitermes schwarzi* [7, 30], and have been tentatively assigned to the genus *Pillotina* [22]. However, the phylogenetic position of these species is not known. In the present study, we documented large spirochetes with ultrastructural features of the genus *Pillotina* in both *K. flavicollis* and *K. italicus* as well as *R. flavipes* (Fig. 2A–C). Individual cells isolated from gut contents of *Kalotermes* spp. fall into cluster B1, which consists exclusively of sequences obtained from this genus (Fig. 1). Although we did not obtain any cells by capillary picking from *R. flavipes* that fell into subcluster B2, this subcluster comprises a sequence from an amplicon library from *R. flavipes* and 16S rRNA gene sequences of two SAGs from *R. speratus*. Since FISH with specific probes against subcluster B2 hybridized large spirochetes in gut homogenates of *R. flavipes* (Fig. 5), we are confident that the phylotypes in subcluster B2 represent the morphotype of *Pillotina* observed in species of the genus *Reticulitermes* in this and in previous studies.

While the phylogenetic distances between representatives of subclusters B1 and B2 are high (85–88% sequence identity) and their sister position lacks bootstrap support (Fig. 1), the monophyly of cluster B is strongly supported in the phylogenomic analysis (Fig. 6). The low relative evolutionary divergence between the genome of *Pillotina corrugata* (subcluster B1) (this study), the two SAGs from *R. speratus* [25] (subcluster B2), and several MAGs from other kalotermitids [15] corroborates that cluster B (g__BOBG01 in GTDB) indeed represents a genus-level lineage (Fig. 6). Although the identity of the type species, *Pillotina calotermitidis*, remains to be established, we assign all representatives of cluster B to the genus *Pillotina* (see Taxonomy section). Notably, we found no evidence for the presence of members of this genus in termites of the genus *Incisitermes*. Phylotypes from cluster B were not represented among the large spirochetal cells isolated from *I. tabogae* and *I.* aff. *schwarzi* and were absent from the amplicon libraries obtained from these and three other species (*I. synderi*, *I. platycephalus,* and *I. incisus*) investigated to date. Moreover, FISH analysis of *I. tabogae* and *I.* aff. *schwarzi* corroborated the absence of large spirochetes other than those assigned to the genus *Hollandina* (see below), and no spirochetes with the ultrastructure of *Pillotina* were detected in the electron micrographs of *I. tabogae*. Therefore, the earlier identification of *Pillotina* in *I. schwarzi* [21] based only on light microscopy is potentially incorrect.

### The genus *Hollandina*

The description of the type species, *Hollandina pterotermitidis*, is based on a morphotype that occurs in the gut of *Pterotermes occidentis* [7, 21]. Similar morphotypes tentatively assigned to the genus *Hollandina* have also been observed in other members of the family Kalotermitidae, including several *Incisitermes* spp., members of the family Rhinotermitidae (*Coptotermes formosanus*, several *Reticulitermes* spp.), the termite *Mastotermes darwiniensis*, and the wood roach *Cryptocercus punctulatus* [7, 21, 28, 35]. Again, no isolates were obtained, and the phylogenetic position of these species remained unknown. In the present study, we documented morphotypes with the ultrastructural features of the genus *Hollandina* in *Kalotermes* spp., *Incisitermes* spp., and *Reticulitermes flavipes* (Fig. 2D–H). The corresponding phylotypes from *Kalotermes* and *Incisitermes* fall into cluster C, which comprises also the 16S rRNA gene sequences from numerous other kalotermitids that were not investigated in the previous studies (Fig. 1). Although the phylogenetic position of the type species remains to be established, we assign all members of cluster C to the genus *Hollandina*. In large-scale amplicon libraries, members of this cluster are present in many kalotermitid hosts but absent from *R. flavipes*, *C. formosanus*, several other rhinotermitids, *M. darwiniensis*, and the wood roach *Cryptocercus punctulatus*, indicating that the large spirochetes with a *Hollandina*-like morphology present in these species belong to lineages outside the radiation of cluster C (Fig. 1, Additional File 1: Fig. S1).

### The genus *Hollandinoides*

Although the sister position of clusters C and D is only poorly supported in the 16S rRNA gene analysis (Fig. 1), the phylogenomic analysis strongly supports that the genomes in cluster D, which consist exclusively of representatives from Rhinotermitidae, represent a genus-level lineage (g__JAITHW01 in GTDB) that is sister to the genus *Hollandina* (Fig. 6). We therefore describe this lineage as a new genus, *Hollandinoides*, with *Hollandinoides gharagozlouae* from *R. flavipes* as the type species.

Our ultrastructural data on the large spirochetes in *R. flavipes* revealed the presence of two distinct *Hollandina-*like morphotypes (Fig. 2G and H). While this is concordant with the presence of two distinct phylotypes in the 16S rRNA gene libraries of this species (clusters D1 and D2; Fig. 1), it is presently not possible to assign a definitive morphotype to *Hollandinoides gharagozlouae*.

### Other large spirochetes

Termites of the genus *Reticulitermes* harbor large spirochetes assigned to the genus *Clevelandina*, whose type species, *Clevelandina reticulitermitidis*, has been described in *R. tibialis* [7]. The morphotypes of *Pillotina* and *Clevelandina* have also been documented in *R. flavipes* [9, 30] and a *Hollandina*-like morphotype in *R. hesperus* [7] (see above). We detected four morphotypes of large spirochetes (Fig. 2C, G-I) in our electron micrographs of *R. flavipes*, which matches the presence of four phylotypes in this species (clusters A, B2, D1, and D2). Although the FISH analysis confirmed that all phylotypes are large spirochetes (Fig. 5), only one morphotype (Fig. 2C) can be firmly linked to the genus *Pillotina* (see above).

While the two *Hollandina*-like morphotypes (Fig. 2G and H) most likely represent *Hollandinoides gharagozlouae* (Cluster D1) and the second, closely related phylotype (cluster D2) from *R. flavipes* in the genus *Hollandoida* (see above), the assignment of the morphotype matching the description of *Clevelandina* (Fig. 2I) remains tentative. Spirochetes with the morphology of *Clevelandina* reportedly occur exclusively in the genus *Reticulitermes* [7], which is consistent with the exclusive presence of representatives from *Reticulitermes* spp. in cluster A, including the remaining, unassigned SCAs and SAGs from *R. flavipes* (Fig. 1). However, in the phylogenomic tree, the SAGs from this cluster form a genus-level clade (g__JAISNI01) that contains also two MAGs from *K. flavicollis* and *I. marginipennis*. While *I. marginipennis* was not investigated in our study, the FISH analysis of *K. flavicollis* showed no spirochetes other than those targeted by the *Pillotina*-specific *and Hollandina-*specific probes. If the morphotype of *Clevelandina* is indeed represented by cluster A, members of this genus may be rare or not consistently present in *K. flavicollis*.

The same argument would explain also the absence of spirochetes with the morphology of *Diplocalyx calotermitidis* from the *K. flavicollis* samples investigated in the present study. The description of this species is based on a morphotype from *Kalotermes* [syn. *Calotermes*, *Incisitermes*] *flavicollis* [7, 20]. It is characterized by the presence of a sillon, a bundle of 70–80 flagella opposing the sillon, an inner coat on the outer membrane, and a thick outer coat on the inner membrane, forming a calyx. However, we did not observe any cells with these unique characteristics in our electron micrographs of *K. flavicollis,* which is consistent with the absence of a third phylotype among the SCAs from this species and corroborated by the results of our FISH analysis of this termite (Fig. 3A). Therefore, we conclude that also *Diplocalyx calotermitidis* is either rare or not consistently present in *K. flavicollis*. In that context, it is important to note that *K. flavicollis* represents a species complex that comprises several regional subgroups, which include the recently described *K. italicus* and at least two distinct lineages of *K. flavicollis* from the Eastern and Western Mediterranean region [36, 37] (Additional File 1: Fig. S2). The provenance of the sample investigated by Gharagozlou [20] is unclear, but it may differ from that of the samples used in our study. A second species, “*Diplocalyx cryptotermitidis*,” has been described based on a morphotype from *Cryptotermes cavifrons* [35]. This species is colonized by numerous phylotypes of *Breznakiellaceae* (Additional File 1: Fig. S1), but the identity and phylogenetic position of the large spirochetes in the genus *Diplocalyx* remain to be investigated.

Nevertheless, the pillotinaceous spirochetes in termite guts are clearly polyphyletic. Since members of *Breznakiellaceae* are generally small and possess only two periplasmic flagella [12], we hypothesize that an increased cell size, flagellar multiplication, and other traits (sillon, decorations of outer and cytoplasmic membranes) must have developed independently and more than once during the evolutionary radiation of the family. This notion is corroborated by the epibiotic spirochetes attached to the gut flagellate *Mixotricha paradoxa* of *Mastotermes darwiniensis*. Here, a smaller, more abundant morphotype, which is responsible for the motility of the flagellate, has only two flagella [38]. It was recently described as *Propulsinema mixotrichae*, and its genome has been sequenced [39]. By contrast, the less abundant forms colonizing the posterior region of the host cell have multiple flagella that are arranged in a single row [38]. Based on their unique morphology, they have been described as “*Canaleparolina darwiniensis*” and were assigned to the “*Pillotinaceae*” even though they are considerably smaller than other species of the family [35]. However, molecular identification revealed that all ectosymbiotic spirochetes of *M. paradoxa* are closely related [40] and fall into a monophyletic clade of *Breznakiellaceae *(cluster M) that occurs exclusively in *Mastotermes darwiniensis* (Additional File 1: Fig. S1). Another example that supports the independent origin of larger cells with multiple flagella within the family is *Helmutkoeniga isoptericolens* from *Incisitermes tabogae*, which possesses eight periplasmic flagella and is significantly larger than all other isolates of *Breznakiellaceae *[41], but is only distantly related to the other lineages of large spirochetes (Fig. 1). Moreover, large pillotinaceous spirochetes were observed only in termites and *Cryptocercus* and are conspicuously absent also from amplicon libraries of other xylophagous insects.

### Functional role of large spirochetes

**Fig. 8:**
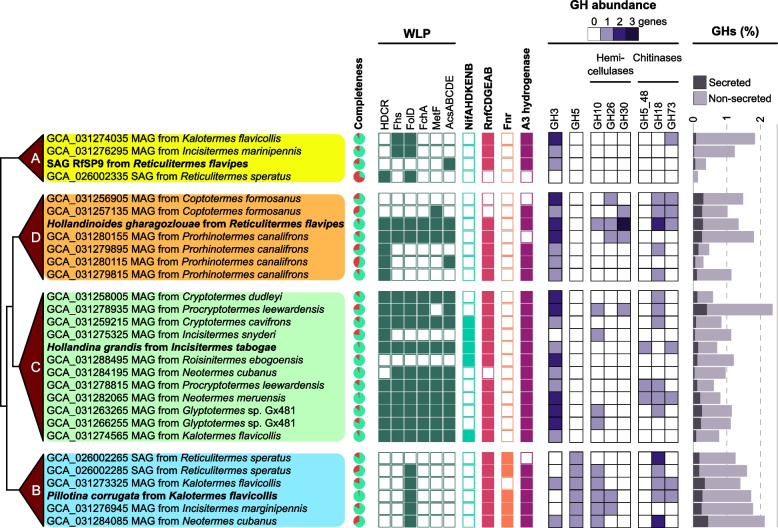
Key enzymes of energy metabolism encoded by MAGs and SAGs in the four genus-level lineages of pillotinaceous spirochetes. Newly sequenced genomes are marked in bold. GH abundance is for secreted enzymes. The phylogenetic tree is schematic and not drawn to scale. Genome completeness is calculated using CheckM2

For a long time, the functional role of spirochetes in termite guts was entirely obscure. Since the isolation of *Treponema primitia*, the first cultured termite gut spirochete and the first homoacetogenic representative of the phylum *Spirochaetota* [42–45], evidence has accumulated that spirochetes are responsible for the high activities of reductive acetogenesis in termite guts (reviewed by [4, 14]). However, most isolates of the *Breznakiellaceae *possess a fermentative metabolism, and the ability to reduce CO_2_ to acetate was most likely acquired by horizontal gene transfer [46]. It is therefore significant that the genomes of *Hollandina grandis* and *Hollandinoides gharagozlouae* encode the complete set of enzymes for an operational WLP (Fig. 7B, C), which indicates the capacity for reductive acetogenesis and the ability to grow homoacetogenically on sugars. Comparative genome analysis of the available metagenome-assembled genomes (MAGs) and SAGs shows that the WLP is likely a common trait to all *Hollandina* species. For *Hollandinoides*, the picture is less clear, as only a few representatives contain the complete WLP (Fig. 8). However, many MAGs of *Hollandinoides* encode the HDCR complex, the key enzyme of reductive acetogenesis in spirochetes, suggesting that the apparent gaps in the pathway are caused by their incomplete genomes (Fig. 8). By contrast, all members of the genera *Pillotina* and JAISNI01 lack key enzymes of the WLP (e.g., HDCR and CODH/ACS) and must grow fermentatively on sugars, forming acetate as the oxidized and H_2_ (and possibly lactate) as the reduced fermentation products (Figs. 7 and 8). The fermentative metabolism of *Pillotina* spp. will yield less ATP than the homoacetogenic metabolism of *Hollandina* and *Hollandinoides* spp., and hydrogen formation by an electron-confurcating hydrogenase may render the fermentative species sensitive to high H_2_ partial pressures [47].

The spectrum of potential substrates differs between the three genera (Fig. 8). While members of all genera possess the capacity to degrade chitin, the genomes of *Pillotina* and *Hollandinoides* species encode several GHs with secretion signals that are most likely involved in the extracellular degradation of hemicelluloses. Moreover, all members of the genus *Pillotina* encode a secreted GH5 endoglucanase involved in cellulose degradation; it is absent from the other lineages. By contrast, the genomes of *Hollandina* and *Hollandinoides* species encode several secreted β-glucosidases that might be involved in the degradation of sugar oligomers.

Generally, *Pillotina and Hollandinoides* spp. encode more GHs than *Hollandina* (Fig. 8)*.* Also the non-secreted GHs, which are mostly involved in the intracellular degradation of oligomeric sugars, are twice as abundant in *P. corrugata* and *Hollandinoides gharagozlouae* than in *H. grandis* (Additional File 2: Table S1, S2, and S3)*.* The situation is similar among the ABC transporters for monomeric and oligomeric sugars (Fig. 7), with the highest number encoded in the genome of *Hollandinoides gharagozlouae* (34 transporters). A large number of ABC transporters in termite gut spirochetes had already been noticed in a broad genomic analysis of the transport proteins in treponemes [48]. The presence of ABC transporters translocating various pentoses corroborates that termite gut spirochetes generally use pentoses as substrates [41, 42, 47].

All species encode several ABC transporters for aldouronates. One of the transporters in *P. corrugata* might be involved in the translocation of aldouronates that result from methylglucuronoxylan depolymerization by the secreted endoxylanase (GH10) (Additional File 2: Table S4). Although not all genes are clustered in an operon, the subsequent intracellular metabolism of aldouronates proceeds via a pathway described in *Paenibacillus* [49, 50]. In *H. grandis*, we identified a gene cluster encoding an aldouronate-like ABC transporter and an unsaturated rhamnogalacturonyl hydrolase (GH105) that resembles an operon in *Bacillus subtilis* involved in the degradation of unsaturated rhamnogalacturonan [51]. In the genome of *Hollandinoides gharagozlouae*, we detected several ABC transporters for rhamnose and pectin oligosaccharides. The capacity to metabolize rhamnose is present in both *H. grandis* and *Hollandinoides gharagozlouae* and seems to proceed via a non-conventional pathway*.* Both genomes lack a typical rhamnulokinase (*rhaB)* but encode a sugar kinase (*rhaK*) similar to a homolog in *Rhizobium leguminosarum*, which requires interaction with the corresponding ABC transporter to phosphorylate rhamnose [52]. Like *Breznakiella homolactica*, *Leadbettera azotonutricia,* and “*Termitinema*” [*Treponema*] *primitia*, also the species investigated in this study possess the entire pathway for the conversion of *N*-acetyl-glucosamine (GlcNAc) to Fru6P but lack a GlcNAc kinase (*nagK*), indicating that GlcNAc metabolism in *Breznakiellaceae *involves an alternative pathway for GlcNAc phosphorylation. A deletion of the *nagK* gene in *Escherichia coli* does not lead to an accumulation of GlcNAc [53].

Compared to other members of the termite gut microbiota, the number of GHs putatively involved in the breakdown of cellulose and hemicellulose in the genomes of *Pillotina, Hollandina, and Hollandinoides* spp*.* is rather small, which agrees with the concept that the spirochetes in lower termites play no major role in the depolymerization of plant fiber [6, 54]. However, the high abundance of ABC transporters for the uptake of monomeric and oligomeric products of fiber digestion suggests that *Pillotina* and *Hollandinoides* spp. are specialized on the metabolism of sugars and dextrins produced by (hemi)cellulolytic flagellates and other gut microbiota. The presence of a WLP and a functional type-III nitrogenase in *H. grandis*, which combines the metabolic capacities of “*Termitinema*” [*Treponema*] *primitia* for reductive acetogenesis [43, 44] and *Leadbettera azotonutricia* for dinitrogen fixation [42, 55], indicates adaptations to the hydrogen-rich gut microenvironment and nitrogen-poor diet of wood-feeding termites [4].

### Taxonomy

*Pillotina calotermitidis* and *Hollandina pterotermitidis* have been described in morphological studies of termite-gut spirochetes [8, 21]. Since the names were not included in the Approved Lists of Bacterial Names [56], they had no standing in nomenclature [Rule 24a, International Code of Nomenclature of Prokaryotes (ICNP); [57]]. However, a few years later, the names were revived and validly published in the *International Journal of Systematic and Evolutionary Microbiology* based on type-descriptive material [7]. The same applies to the species descriptions of *Diplocalyx calotermitidis* and *Clevelandina reticulitermitidis* [7, 20]. Subsequent species descriptions of other large termite-gut spirochetes (“*Diplocalyx cryptotermitidis”* and “*Canaleparolina darwiniensis”*; [35]) were published after January 1, 1980 and—in the absence of type strains—these names are not validly published under the rules of the ICNP. The classification of the genera *Clevelandina*, *Diplocalyx*, *Hollandina*, and *Pillotina* in the family “*Pillotinaceae*” [8, 22] was superseded by their placement in the family *Spirochaetaceae *[58]. Although the latter study acknowledged the lack of 16S rRNA gene sequence data for all representatives of these taxa, a rationale for this placement and the rejection of the family “*Pillotinaceae*” was not provided.

In the present study, we identified three phylogenetic clades of large spirochetes with the morphology of *Pillotina* and *Hollandina* spp. from several lower termites. Using high-quality genome sequences obtained from single cells as type material, we describe *Pillotina corrugata* sp. nov. from *K. flavicollis, Hollandina grandis* sp. nov. from *I. tabogae,* and *Hollandinoides gharagozlouae* gen. nov., sp. nov. from *R. flavipes* under the rules of SeqCode [27]. The protologues can be found in the supplementary material (Additional File 3: Table S7). In addition, we propose reassigning members of the genera *Pillotina* and *Hollandina* that are currently assigned to *Spirochaetaceae *to the family *Breznakiellaceae*. While it is very likely that also members of *Clevelandina*, *Diplocalyx*, and “*Canaleparolina*” are members of *Breznakiellaceae*, their formal reassignment should be postponed until they have been firmly assigned to specific phylotypes with sequenced genomes.

#### *Breznakiellaceae *Brune et al. 2022 emend. Treitli and Brune

The description remains the same as that in Brune et al. (2022), with the following amendments:

Large forms are up to 100 μm long and 0.4–1.5 μm in diameter and possess 15–100 flagella. GC content is 43.2–52.9 mol% (based on sequenced genomes). In addition to the genera *Breznakiella*, *Gracilinema*, *Helmutkoenigia*, *Leadbettera,* and *Zuelzera*, the family also comprises the newly described genus *Hollandinoides* and the genera *Pillotina* and *Hollandina*, which were previously classified in the families “*Pillotinaceae*” and *Spirochaetaceae*.

**Parent taxon:** “*Treponematales”.*

#### *Pillotina* (ex Hollande and Gharagozlou 1968) Bermudes et al. 1988 emend. Treitli and Brune

*Pillotina* (Pil.lo.ti'na. M.L. fem. n. *Pillotina*, in honor of J. Pillot, a French microbiologist). Helical cells are 0.6–1.5 μm in diameter. Transverse sections show a stellate profile with approximately 30–100 flagella distributed throughout the periplasmic space. The outer membrane is crenulated (pleated or folded) and has an underlying electron-dense layer. One of the grooves is in contact with the cytoplasmic cylinder, forming the sillon. They are anaerobes with a strictly fermentative metabolism (based on genome analysis of *P. corrugata*). Members of the genus are defined by phylogenomic analysis as a monophyletic group that shows a relative evolutionary divergence (RED) similar to that of the neighboring genera. The description of the type species is based on a morphotype from the termite *Postelectrotermes praecox*. The only species with a sequenced genome is *Pillotina corrugata*.

**Type species:** *Pillotina calotermitidis.*

**Parent taxon:** *Breznakiellaceae*.

#### *Pillotina corrugata* sp. nov. Treitli and Brune

*Pillotina corrugata* (cor.ru.ga'ta). L. fem. part. adj. *corrugata*, wrinkled, corrugated. Helical cells are 0.9–1.2 µm in diameter, with 26–30 parallel ridges. The length varies between 40 and 90 µm. Approx. 70–100 periplasmic flagella. The protoplasmic cylinder is 0.4–0.5 µm in diameter. Members of this species colonize the hindgut of the genus *Kalotermes*. They can be detected with the 16S rRNA-targeted oligonucleotide probe Kf-Pil-1449 (5′-GCAGCGCCCTCCTTTTACAA-3′). The type genome is from *Kalotermes flavicollis*.

Includes all genomes that show ≥ 95% average nucleotide identity (ANI) to the genome of the type strain. The type strain has an estimated genome size of 3.14 Mbp and a GC content of 43.2 mol%.

**Type genome:** KfSPG140; JBQMQA000000000, PX098836 (16S rRNA gene).

#### *Hollandina* (ex To et al. 1978) Bermudes et al. 1988 emend. Treitli and Brune

*Hollandina* (Hol.lan.di'na. M.L. fem. n. *Hollandina*, in honor of André Hollande, Jr., a French protistologist). Helical cells are rounded to oblong in cross sections, with a diameter of 0.4–0.9 µm at the widest point. Cells have 15–80 flagella distributed in the periplasmic space. The outer membrane is smooth and decorated with a thick external coat, in some cases forming a sillon. In cross sections, the outer membrane is in close proximity to the cytoplasmic membrane over about half of its circumference, restricting the distribution of the flagella to the remaining periplasmic space. Anaerobes with a fermentative metabolism (based on genome analysis of *H. grandis*). The genus is defined by phylogenomic analysis as a monophyletic group that shows a relative evolutionary divergence (RED) similar to that of the neighboring genera. Members of the genus occur in termites of the family Kalotermitidae. The description of the type species is based on a morphotype from the termite *Pterotermes occidentis*. The only species with a sequenced genome is *Hollandina grandis*.

**Type species:** *Hollandina pterotermitidis.*

**Parent taxon:** *Breznakiellaceae*.

#### *Hollandina grandis* sp. nov. Treitli and Brune

*Hollandina grandis* (gran'dis). L. fem. adj. *grandis*, large. Helical cells are rounded to oblong in cross sections, with a diameter of 0.5–0.7 µm at the widest point. The length varies between 30 and 80 µm. Approx. 70–80 periplasmic flagella. The rounded protoplasmic cylinder is 0.4–0.5 µm in diameter. Members of this species colonize the hindgut of the genus *Incisitermes*. They can be detected with the 16S rRNA-targeted oligonucleotide probe It-Hol-183 (5′-CCATGCCACAGCACGATAAG-3′). The type genome is from the hindgut of the termite *Incisitermes tabogae*.

Includes all genomes that show ≥ 95% average nucleotide identity (ANI) to the type genome. The type genome has an estimated size of 3.72 Mbp and a GC content of 49.1 mol%.

**Type genome:** ItSP2; JBQLJB000000000; PX098833 (16S rRNA gene).

#### “*Candidatus* Hollandina kalotermitidis” sp. nov. Treitli and Brune

*Candidatus* Hollandina kalotermitidis (ka.lo.ter.mi'ti.dis) M.L. gen. n. *kalotermitidis*, of *Kalotermes*, a genus of termites. Helical cells are rounded to oblong in cross section, with a diameter of 0.7–0.8 µm at the widest point. The length varies between 20 and 50 µm. Approx. 40–60 periplasmic flagella. The rounded protoplasmic cylinder is 0.5–0.6 µm in diameter.

Members of this species are present in *Kalotermes flavicollis* and *K. italicus*. They can be detected with the 16S rRNA-targeted oligonucleotide probe Kf-Hol-193 (5′-GAGCCACAGCCCCTTTCCT-3′). Type material is picked from cells of *K. flavicollis* (PX098799; 16S rRNA gene).

#### *Hollandinoides* gen. nov. Treitli and Brune

*Hollandinoides* (Hol.lan.di.no'i.des. N.L. neut. n. *Hollandina*, a genus of spirochetes; L. neut. suff. *-oides*, -like, similar; N.L. neut. n. *Hollandinoides*, a *Hollandina*-like genus).

The description is the same as that of the type species*.*

**Type species:** *Hollandinoides gharagozlouae.*

**Parent taxon** ***:*** *Breznakiellaceae.*

#### *Hollandinoides gharagozlouae* sp. nov. Treitli and Brune

*Hollandinoides gharagozlouae* (gha.ra.goz'lou.ae). N.L. gen. fem. n. *gharagozlouae*, in honor of Iran Dokht Gharagozlou, an Iranian/French structural biologist. Large helical cells that colonize the hindgut of the termite *Reticulitermes flavipes*. They can be detected with the 16S rRNA-targeted oligonucleotide probe Rf-HolD1-990 (5'-GGCTTCCCCACTATGTCAAA-3'). The type genome is from the hindgut of the termite *Reticulitermes flavipes*. The species is most likely represented by one of the two *Hollandina*-like morphotypes present in this host species.

Includes all genomes that show ≥ 95% average nucleotide identity (ANI) to the type genome. The type genome has an estimated size of 4.02 Mbp and a GC content of 47 mol%.

**Type genome:** RfSP5; JBUAPY000000000; PX098808 (16S rRNA gene).

## Conclusions

Using single-cell techniques, we provide the first insights into the evolutionary relationships and metabolic functions of the large spirochetes in termite guts. Phylogenetic analysis shows that “*Pillotinaceae*” are not a separate family of spirochetes but represent several distinct lineages in the family *Breznakiellaceae*. The polyphyletic nature of these lineages suggests that an increased cell size and flagellar multiplication are convergent traits that evolved several times within the family. Comparative analysis of the single-cell genomes of *P. corrugata*, *H. grandis*, and *Hollandinoides gharagozlouae* documents differences in their metabolic potential, indicating that the large spirochetes in termite guts have distinct roles in symbiotic digestion.

## Methods

### Sample preparation for FISH

*Kalotermes flavicollis*, *Incisitermes* aff. *schwarzi,* and *Reticulitermes flavipes* were from cultures maintained at the Federal Institute for Materials Research and Testing (BAM), Berlin, Germany. *Kalotermes italicus* was collected in Tuscany, Italy. *Incisitermes tabogae* was collected in Guadeloupe, French West Indies. To remove most autofluorescent wood particles from the hindgut, several worker termites were placed on cellulose powder for one week prior to dissection [59]. Afterwards, the workers were dissected and the entire hindgut was placed in solution U [60] and opened with fine-tipped tweezers. The gut content was mixed with three volumes of ice-cold 4% formaldehyde, and fixed on ice for 6 h. After fixation, cells were pelleted at three different speeds. The large protist cells were pelleted at 50 × *g* for 3 min. The supernatant from this centrifugation was transferred to a new tube and centrifuged at 1000 × *g* for 10 min at 4 °C. The supernatant from the second centrifugation was transferred to a new tube and centrifuged at 6000 × *g* for 5 min at 4 °C. This technique allows harvesting of most of the intestinal community without breaking the cells. The pellets resulting from the centrifugations were washed three times with solution U. After the final wash, each pellet was resuspended in solution U, mixed with an equal volume of ethanol, and stored at − 20 °C. Samples sedimented by centrifugation at 1000 × *g* and 6000 × *g* were used for FISH.

### Probe design for 16S rRNA FISH

The 16S rRNA gene sequences obtained by PCR as well as the homologs identified in our sequenced single-cell genomes were imported into an in-house database of bacterial 16S rRNA genes, which is based on the phylogenetic framework of Silva v.138.1 [61] and includes sequences extracted from metagenomic libraries and MAGs and previously unpublished clone libraries and long-read amplicon libraries of arthropod gut microbiota obtained in our laboratory [62] (Additional File 2: Table S8). For probe design, we used ARB v. 7.1 [63] (http://www.arb-home.de). The probes suggested by ARB were checked for accessibility based on the *Escherichia coli* 16S rRNA gene accessibility map [64]. The selected probes were synthesized and fluorescently labeled at their 5′ end with either CY3 or various ATTO dyes by Eurofins Genomics (Germany). Probe sequences and their optimal formamide concentrations are listed in Table 1.

### *Fluorescence *in situ* hybridization*

Samples for FISH were prepared according to the protocol described in [65]. Hybridizations were carried out at 46 °C for 6 h with 30% formamide, unless the probes required lower concentration (Table 1). Slides were mounted with Vectashield mounting medium with DAPI (H-1200, Vector Laboratories). Images were acquired using a Leica SP8 confocal microscope, deconvolved using Huygens Essential v17.04 (Scientific Volume Imaging), and further processed using ImageJ 1.53n (https://imagej.net).

### Single-cell picking and whole genome amplification

For single-cell picking, the gut of a worker termite was removed and opened in solution U, and aliquots (10 µl) were transferred into the wells of a Teflon-coated microscope slide (1216690, Marienfeld). Large spirochetes were identified by morphology using an inverted phase-contrast microscope (Zeiss AxioVert A1) at 400-fold magnification and isolated with a PatchMan NP2 micromanipulator (Eppendorf) equipped with a 15-µm capillary. Individual cells were washed twice in solution U and then transferred to a 0.2-ml PCR tube containing 0.9 µl of solution U. Whole genome amplification (WGA) was performed using the Repli-G Single Cell Kit (150343, QIAGEN) with 30% of the recommended reaction volume and adding SYBR green to a final concentration of 1 µM. Briefly, 0.9 µl of lysis buffer was added to the picked cells, and the reaction was incubated for 10 min on ice. Then the lysis was stopped by adding 0.9 µl of stop solution. To the isolated DNA, 8.7 µl of reaction buffer, 0.6 µl of polymerase, 1.5 µl of 10 µM SYBR green solution, and 1.2 µl of water were added. The tubes were incubated at 30 °C in a CFX Connect real-time PCR cycler (Bio-Rad), with fluorescence readings every 5 min. After the amplification reactions reached a plateau, the samples were further incubated at 30 °C for 50 min, and then the reactions were stopped by incubating the samples at 65 °C for 10 min. The whole-genome-amplified DNA was purified by ethanol precipitation, and DNA concentration was measured using Qbit dsDNA Quantitation, Broad Range Kit (Q32853, ThermoFisher).

### Amplification of 16S rRNA gene sequences

16S rRNA genes were amplified using the universal bacterial primers 9/27F and 1492R [66] in 50 µL reactions using PrimeSTAR Max DNA polymerase premix (R045A, Takara Bio). The PCR conditions were set according to the manufacturer’s recommendations, with an annealing temperature of 60 °C for 15 s and extension at 72 °C for 30 s. The amplification products were purified using the Bio-On-Magnetic-Beads (BOMB) platform protocols [67] and sequenced in both directions at Eurofins Genomics (Germany).

### Electron microscopy

For each species, the guts of 10 worker termites were removed and opened in a fixative containing 2.5% glutaraldehyde in 0.1 M sodium cacodylate buffer (pH 7.1). The gut contents were centrifuged at 50 × *g* for 3 min in a swing-out rotor (Eppendorf) to remove most of the termite gut flagellates. The supernatant was transferred to a new tube and centrifuged at 10,000 × *g* for 5 min**.** After removing the supernatant, the pellet was washed three times with 0.1 M cacodylate buffer, postfixed in 1% OsO_4_ for 1 h, washed three times with 0.1 M cacodylate buffer, dehydrated in an ethanol series (30%, 50%, 70%, 90%; 4 times 100%; 15 min each), and embedded in Spurr’s resin [68]. Ultrathin sections were cut with a Reichert Ultracut E ultramicrotome. The sections were stained with saturated uranyl acetate and lead citrate [69] and examined with a FEI CM120 BioTwin electron microscope (FEI) or a FEI Tecnai Spirit Twin 120 kV (FEI).

### Genome sequencing, assembly, and binning

The WGA products from isolated *Pillotina* cells were sequenced at Novogene (Germany), using an Illumina NovaSeq 6000 platform with 2 × 150 bp reads. The WGA products from isolated *Hollandina* cells were sequenced at the Göttingen Genomics Laboratory, Institute of Microbiology and Genetics, Georg August University, Göttingen using an Illumina MiSeq platform with 2 × 300 bp reads. The WGA products from isolated cells from *R. flavipes* were sequenced at the Biocev Core Facility (OMICS-Genomics, Biocev, Vestec, Czech Republic) using an Illumina MiSeq platform with 2 × 250 bp reads. Raw sequencing reads were adapter-trimmed and quality-trimmed using Fastp [70] with a quality threshold of 15. Each individual single-cell genome was assembled using SPAdes 3.15.5 [71] with the –sc parameter. The 16S rRNA gene sequences of each dataset were extracted, and samples with identical 16S rRNA gene sequences were grouped and co-assembled with SPAdes.

Scaffolds longer than 2500 bp were binned using tetraESOM [72]. The resulting bins were checked for contamination using blastn and blastp; sequences with significant hits to other bacteria were removed. The final bins were reassembled using SPAdes by mapping back the Illumina reads using BBMap and discarding any unmapped reads. After reassembly, fragments less than 1000 bp were removed, and the genome was checked again for potential contamination using a combination of blastn and blastp.

### Genome completeness estimation and annotation

Genome completeness was estimated using both CheckM [73] and CheckM2 [74]. Genomes were predicted and annotated using Prokka 1.14.6 [75]. Annotations were further refined using BlastKOALA [76] and KofamKOALA [77]. For the annotation of carbohydrate-active enzymes (CAZymes), we used the dbCAN3 server for Hidden Markov model (HMM) searches and DIAMOND blast searches [78] against the dbCAN3 database [79]. For all pathways of interest, the annotation was manually curated.

### Comparative genomics

A total of 29 MAGs and SAGs were selected for comparative genomics. The predicted protein sequences from all the genomes were downloaded from NCBI and clustered into orthogroups using OrthoFinder2 [80]. The obtained hierarchical orthogroups (HOGs) were used for further annotation. The annotated genes from *Hollandina* and *Pillotina* were used as seeds to identify the HOGs with specific functions. For the identification of genes involved in nitrogen fixation, we identified each gene by blastp and checked the operon structure to make sure that all genes are present in a functional operon. For the annotation of carbohydrate-active enzymes (CAZymes), we used dbCAN3 with SignalP v4.1 [81].

### Phylogenetic analyses

The 16S rRNA gene sequences obtained in this study were imported into the same in-house database as that used for probe design (see above). Sequences were aligned using the SINA aligner [82] and ARB software package version 7.1 [63]. After dereplication, alignment, and filtering, the final dataset of *Spirochaetota* contained 513 sequences with 1,470 alignment positions. It was used to compute a maximum-likelihood (ML) tree with IQ-TREE 3.0.1 [83] and the GTR + G + F + I model. Branch support was assessed using SH-aLRT and 10,000 ultrafast bootstrap replicates.

The dataset for phylogenomic analysis was created using GTDB-Tk [84] with the GTDB database version 220 [85]. The phylogenomic tree was inferred using IQ-TREE 3.01 with the Posterior Mean Site Frequency (PMSF) empirical model [86] and an LG + F + G guide tree. Branch support was estimated using SH-aLRT and 10,000 ultrafast bootstrap replicates.

## Supplementary Information

Additiopnal file 1. Supplementary figures S1-S2. Fig. S1 – Expanded 16S rRNA gene-based maximum-likelihood (ML) tree of the phylum *Spirochatetota*. Fig. S2 – Phylogeny of the *Kalotermes* termites based on cytochrome-c oxidase subunit II.

Additional file 2. Supplementary tables S1-S6, S8. Table S1 – Glycoside hydrolase families identified in the genome of *Pillotina corrugata*. Table S2 – Glycoside hydrolase families identified in the genome of *Hollandina grandis*. Table S3 – Glycoside hydrolase families identified in the genome of *Hollandinoides gharagozlouae*. Table S4 – Gene annotation list for *Pillotina corrugata*. Table S5 – Gene annotation list for *Hollandina grandis*. Table S6 – Gene annotation list for *Hollandinoides gharagozlouae*.  Table S8 – 16S rRNA gene libraries inspected for the presence of *Breznakiellaceae*, with the accession numbers for the identified *Breznakiellaceae* sequences*.*

Additional file 3. Supplementary table S7. Table S7 – Protologues for the new species described under SeqCode.

## Data Availability

The sequence data have been deposited at the National Center for Biotechnology Information (NCBI) in GenBank (https://www.ncbi.nlm.nih.gov/genbank) under NCBI BioProject PRJNA1301810. The scaffolds assigned to the draft genomes of *Pillotina corrugata*, *Hollandina grandis*, and *Hollandinoides gharagozlouae* have been deposited at GenBank under the accession nos. JBQMQA000000000, JBQLJB000000000, and JBUAPY000000000, respectively). The genome assembly of the RfSP9 SAG was deposited at GenBank under the accession number JBTZSX000000000. Newly obtained 16S rRNA gene sequences are available in GenBank under accession numbers PX098795–PX098819, and PX098832–PX098836.

## Abbreviations

EM: Electron microscopy
FISH: Fluorescence in situ hybridization
GH: Glycosyl hydrolases
SAG: Single-cell amplified genome
SCA: Single-cell amplicon
WGA: Whole-genome amplification
WLP: Wood–Ljungdahl pathway

## References

[1] Breznak JA, Leadbetter JR. Termite gut spirochetes. In: Dworkin M, Falkow S, Rosenberg E, Schleifer K-H, Stackebrandt E, editors. The Prokaryotes: Volume 7: Proteobacteria: Delta, Epsilon Subclass. New York: Springer; 2006;318–29. 10.1007/0-387-30747-8_11.

[2] Brune A, Dietrich C. The gut microbiota of termites: digesting the diversity in the light of ecology and evolution. Annu Rev Microbiol. 2015;69:145–66. 10.1146/annurev-micro-092412-155715. 26195303 10.1146/annurev-micro-092412-155715

[3] Ohkuma M. Symbioses of flagellates and prokaryotes in the gut of lower termites. Trends Microbiol. 2008;16:345–52. 10.1016/j.tim.2008.04.004. 18513972 10.1016/j.tim.2008.04.004

[4] Brune A. Symbiotic digestion of lignocellulose in termite guts. Nat Rev Microbiol. 2014;12:168–80. 10.1038/nrmicro3182. 24487819 10.1038/nrmicro3182

[5] Hervé V, Liu P, Dietrich C, Sillam-Dussès D, Stiblik P, Šobotník J, et al. Phylogenomic analysis of 589 metagenome-assembled genomes encompassing all major prokaryotic lineages from the gut of higher termites. PeerJ. 2020;2020:e8614. 10.7717/PEERJ.8614/SUPP-20. 10.7717/peerj.8614 PMC7024585 32095380

[6] Tokuda G, Mikaelyan A, Fukui C, Matsuura Y, Watanabe H, Fujishima M, et al. Fiber-associated spirochetes are major agents of hemicellulose degradation in the hindgut of wood-feeding higher termites. Proc Natl Acad Sci U S A. 2018;115:E11996. 10.1073/pnas.1810550115. 30504145 10.1073/pnas.1810550115 PMC6304966

[7] Bermudes D, Chase D, Margulis L. Morphology as a basis for taxonomy of large spirochetes symbiotic in wood-eating cockroaches and termites: *Pillotina* gen. nov., nom. rev.; *Pillotina calotermitidis* sp. nov., nom. rev.; *Diplocalyx* gen. nov., nom. rev.; *Diplocalyx calotermitidis* sp. nov., nom. rev.; *Hollandina* gen. nov., nom. rev.; *Hollandina pterotermitidis* sp. nov., nom. rev.; and *Clevelandina reticulitermitidis* gen. nov., sp. nov. Int J Syst Evol Microbiol. 1988;38:291–302. 10.1099/00207713-38-3-291. 10.1099/00207713-38-3-291 11542253

[8] Hollande A, Gharagozlou I. Morphologie infrastructurale de *Pillotina calotermitidis* nov. gen., nov. sp., Spirochaetale de l’intestin de *Calotermes praecox*. Comptes Rendus Hebd Seances Acad Sci Ser Sci Nat. 1967;265:1309–12. 4967239

[9] Paster BJ, Breznak JA. Hindgut spirochetes of termites and *Cryptocercus punctulatus*. In: Trujillo ME, Dedysh S, DeVos P, Hedlund B, Kämpfer K, Rainey FA, editors. Bergey’s manual of systematics of Archaea and Bacteria. John Wiley & Sons, Ltd; 2015. p. 1–5. 10.1002/9781118960608.fbm00242.

[10] Lilburn TG, Schmidt TM, Breznak JA. Phylogenetic diversity of termite gut spirochaetes. Environ Microbiol. 1999;1:331–45. 10.1046/j.1462-2920.1999.00043.x. 11207751 10.1046/j.1462-2920.1999.00043.x

[11] Ohkuma M, Iida T, Kudo T. Phylogenetic relationships of symbiotic spirochetes in the gut of diverse termites. FEMS Microbiol Lett. 1999;181:123–9. 10.1111/j.1574-6968.1999.tb08834.x. 10564797 10.1111/j.1574-6968.1999.tb08834.x

[12] Brune A, Song Y, Oren A, Paster BJ. A new family for ‘termite gut treponemes’: description of *Breznakiellaceae *fam. nov., *Gracilinema caldarium* gen. nov., comb. nov., *Leadbettera azotonutricia* gen. nov., comb. nov., *Helmutkoenigia isoptericolens* gen. nov., comb. nov., and *Zuelzera stenostrepta* gen. nov., comb. nov., and proposal of *Rectinemataceae* fam. nov. Int J Syst Evol Microbiol. 2022;72:005439. 10.1099/ijsem.0.005439. 10.1099/ijsem.0.005439 35639582

[13] Noda S, Shimizu D, Yuki M, Kitade O, Ohkuma M. Host-symbiont cospeciation of termite-gut cellulolytic protists of the genera *Teranympha* and *Eucomonympha* and their *Treponema* endosymbionts. Microbes Environ. 2018;33:26–33. 10.1264/jsme2.ME17096. 29367472 10.1264/jsme2.ME17096 PMC5877339

[14] Ohkuma M, Noda S, Hattori S, Iida T, Yuki M, Starns D, et al. Acetogenesis from H_2_ plus CO_2_ and nitrogen fixation by an endosymbiotic spirochete of a termite-gut cellulolytic protist. Proc Natl Acad Sci U S A. 2015;112:10224–30. 10.1073/pnas.1423979112. 25979941 10.1073/pnas.1423979112 PMC4547241

[15] Mies US, Hervé V, Kropp T, Platt K, Sillam-Dussès D, Šobotník J, et al. Genome reduction and horizontal gene transfer in the evolution of *Endomicrobia*—rise and fall of an intracellular symbiosis with termite gut flagellates. mBio. 2024;15:e00826-24. 10.1128/mbio.00826-24. 38742878 10.1128/mbio.00826-24 PMC11257099

[16] Dröge S, Fröhlich J, Radek R, König H. *Spirochaeta coccoides* sp. nov., a novel coccoid spirochete from the hindgut of the termite *Neotermes castaneus*. Appl Environ Microbiol. 2006;72:392–7. 10.1128/AEM.72.1.392-397.2006. 16391069 10.1128/AEM.72.1.392-397.2006 PMC1352290

[17] Sravanthi T, Tushar L, Sasikala C, Ramana C. *Alkalispirochaeta cellulosivorans* gen. nov., sp. nov., a cellulose-hydrolysing, alkaliphilic, halotolerant bacterium isolated from the gut of a wood-eating cockroach (*Cryptocercus punctulatus*), and reclassification of four species of *Spirochaeta* as new combinations within *Alkalispirochaeta* gen. nov. Int J Syst Evol Microbiol. 2016;66:1612–9. 10.1099/ijsem.0.000865. 26704619 10.1099/ijsem.0.000865

[18] Leidy J. On intestinal parasites of *Termes flavipes*. Proc Acad Nat Sci Phila. 1877;29:146–9.

[19] Leidy J. The parasites of the termites. J Acad Nat Sci Phila. 1881;VIII:425–47.

[20] Gharagozlou ID. Aspect infrastructural de *Diplocalyx calotermitidis* nov. gen. nov. sp., Spirochaetale de l’intestin de *Calotermes flavicollis*. Comptes Rendus Hebd Seances Acad Sci Ser Sci Nat. 1968;266:494–6. 4967239

[21] To L, Margulis L, Cheung AT. Pillotinas and hollandinas: distribution and behaviour of large spirochaetes symbiotic in termites. Microbios. 1978;22:103–33. 753948

[22] Margulis L, Hinkle G. Large symbiotic spirochetes: *Clevelandina*, *Cristispira*, *Diplocalyx*, *Hollandina*, and *Pillotina*. In: Balows A, Trüper HG, Dworkin M, Harder W, Schleifer K-H, editors. The prokaryotes: a handbook on the biology of bacteria: ecophysiology, isolation, identification, applications. New York, NY: Springer; 1992;3965–78. 10.1007/978-1-4757-2191-1_59.

[23] Leschine S, Paster BJ, Canale-Parola E. Free-living saccharolytic spirochetes: the genus *Spirochaeta*. In: Dworkin M, Falkow S, Rosenberg E, Schleifer K-H, Stackebrandt E, editors. The prokaryotes: Volume 7: Proteobacteria: Delta, Epsilon Subclass. New York, NY: Springer; 2006;195–210. 10.1007/0-387-30747-8_7.

[24] Nakamura S. Spirochete flagella and motility. Biomolecules. 2020;10:550. 10.3390/biom10040550. 32260454 10.3390/biom10040550 PMC7225975

[25] Aoki H, Masahiro Y, Shimizu M, Hongoh Y, Ohkuma M, Yamagata Y. Agarose gel microcapsules enable easy-to-prepare, picolitre-scale, single-cell genomics, yielding high-coverage genome sequences. Sci Rep. 2022;12:17014. 10.1038/s41598-022-20923-z. 36257967 10.1038/s41598-022-20923-z PMC9579161

[26] Arora J, Kinjo Y, Šobotník J, Buček A, Clitheroe C, Stiblik P, et al. The functional evolution of termite gut microbiota. Microbiome. 2022;10:78. 10.1186/s40168-022-01258-3. 35624491 10.1186/s40168-022-01258-3 PMC9137090

[27] Hedlund BP, Chuvochina M, Hugenholtz P, Konstantinidis KT, Murray AE, Palmer M, et al. SeqCode: a nomenclatural code for prokaryotes described from sequence data. Nat Microbiol. 2022;7:1702–8. 10.1038/s41564-022-01214-9. 36123442 10.1038/s41564-022-01214-9 PMC9519449

[28] Margulis L, To LP, Chase DG. The genera *Pillotina*, *Hollandina*, and *Diplocalyx*. In: Starr MP, Stolp H, Trüper HG, Balows A, Schlegel HG, editors. The prokaryotes: a handbook on habitats, isolation, and identification of Bacteria. Berlin, Heidelberg: Springer Berlin Heidelberg; 1981;548–54. 10.1007/978-3-662-13187-9_47.

[29] Grimstone AV. A note on the fine structure of a spirochaete. J Cell Sci. 1963;s3-104:145–53. 10.1242/jcs.s3-104.65.145.

[30] Breznak JA. Hindgut spirochetes of termites and *Cryptocercus punctulatus*. In: Krieg NR, Holt JG, editors. Bergey’s manual of systematic bacteriology, vol. 1. Baltimore: Williams & Wilkins; 1984. p. 67–70.

[31] Hongoh Y, Deevong P, Hattori S, Inoue T, Noda S, Noparatnaraporn N, et al. Phylogenetic diversity, localization, and cell morphologies of members of the Candidate phylum TG3 and a subphylum in the phylum *Fibrobacteres*, recently discovered bacterial groups dominant in termite guts. Appl Environ Microbiol. 2006;72:6780–8. 10.1128/AEM.00891-06. 17021231 10.1128/AEM.00891-06 PMC1610327

[32] Ochiai A, Itoh T, Kawamata A, Hashimoto W, Murata K. Plant cell wall degradation by saprophytic *Bacillus subtilis* strains: gene clusters responsible for rhamnogalacturonan depolymerization. Appl Environ Microbiol. 2007;73:3803–13. 10.1128/AEM.00147-07. 17449691 10.1128/AEM.00147-07 PMC1932723

[33] Dos Santos PC, Fang Z, Mason SW, Setubal JC, Dixon R. Distribution of nitrogen fixation and nitrogenase-like sequences amongst microbial genomes. BMC Genomics. 2012;13:162. 10.1186/1471-2164-13-162. 22554235 10.1186/1471-2164-13-162 PMC3464626

[34] Morimoto Y, Uesaka K, Fujita Y, Yamamoto H. A nitrogenase-like enzyme is involved in the novel anaerobic assimilation pathway of a sulfonate, isethionate, in the photosynthetic bacterium *Rhodobacter capsulatus*. mSphere. 2024;9:e00498-24. 10.1128/msphere.00498-24. 39191391 10.1128/msphere.00498-24 PMC11423573

[35] Wier A, Ashen J, Margulis L. *Canaleparolina darwiniensis*, gen. nov., sp. nov., and other pillotinaceous spirochetes from insects. Int Microbiol. 2000;3:213–23. 11334304

[36] Ghesini S, Marini M. A dark-necked drywood termite (Isoptera: Kalotermitidae) in Italy: description of *Kalotermes italicus* sp. nov. Fla Entomol. 2013;96:200–11. 10.1653/024.096.0127.

[37] Velonà A, Luchetti A, Ghesini S, Marini M, Mantovani B. Mitochondrial and nuclear markers highlight the biodiversity of *Kalotermes flavicollis* (Fabricius, 1793) (Insecta, Isoptera, Kalotermitidae) in the Mediterranean area. Bull Entomol Res. 2011;101:353–64. 10.1017/S000748531000060X. 21226979 10.1017/S000748531000060X

[38] Cleveland LR, Grimstone AV. The fine structure of the flagellate *Mixotricha paradoxa* and its associated micro-organisms. Proc R Soc Lond B Biol Sci. 1964;159:668–86. 10.1098/rspb.1964.0025.

[39] Fu J, Liu Y, Yoshioka T, Igai K, Mabuchi T, Kihara K, et al. Functional division of labor in motility, lignocellulose digestion, and nitrogen metabolism revealed for the *Mixotricha paradoxa* holobiont. ISME J. 2025;wraf178. 10.1093/ismejo/wraf178. 40832871 10.1093/ismejo/wraf178 PMC12483993

[40] Wenzel M, Radek R, Brugerolle G, König H. Identification of the ectosymbiotic bacteria of *Mixotricha paradoxa* involved in movement symbiosis. Eur J Protistol. 2003;39:11–23. 10.1078/0932-4739-00893.

[41] Dröge S, Rachel R, Radek R, König H. *Treponema isoptericolens* sp. nov., a novel spirochaete from the hindgut of the termite *Incisitermes tabogae*. Int J Syst Evol Microbiol. 2008;58:1079–83. 10.1099/ijs.0.64699-0. 18450692 10.1099/ijs.0.64699-0

[42] Graber JR, Leadbetter JR, Breznak JA. Description of *Treponema azotonutricium* sp. nov. and *Treponema primitia* sp. nov., the first spirochetes isolated from termite guts. Appl Environ Microbiol. 2004;70:1315–20. 10.1128/AEM.70.3.1315-1320.2004. 15006748 10.1128/AEM.70.3.1315-1320.2004 PMC368361

[43] Graber JR, Breznak JA. Physiology and nutrition of *Treponema primitia*, an H_2_/ CO_2_-acetogenic spirochete from termite hindguts. Appl Environ Microbiol. 2004;70:1307–14. 10.1128/AEM.70.3.1307-1314.2004. 15006747 10.1128/AEM.70.3.1307-1314.2004 PMC368360

[44] Leadbetter JR, Schmidt TM, Graber JR, Breznak JA. Acetogenesis from H_2_ plus CO_2_ by spirochetes from termite guts. Science. 1999;283:686–9. 10.1126/science.283.5402.686. 9924028 10.1126/science.283.5402.686

[45] Rosenthal AZ, Zhang X, Lucey KS, Ottesen EA, Trivedi V, Choi HMT, et al. Localizing transcripts to single cells suggests an important role of uncultured deltaproteobacteria in the termite gut hydrogen economy. Proc Natl Acad Sci U S A. 2013;110:16163–8. 10.1073/pnas.1307876110. 24043823 10.1073/pnas.1307876110 PMC3791709

[46] Song Y, Hervé V, Radek R, Pfeiffer F, Zheng H, Brune A. Characterization and phylogenomic analysis of *Breznakiella homolactica* gen. nov. sp. nov. indicate that termite gut treponemes evolved from non-acetogenic spirochetes in cockroaches. Environ Microbiol. 2021;23:4228–45. 10.1111/1462-2920.15600. 33998119 10.1111/1462-2920.15600

[47] Song Y, Pfeiffer F, Radek R, Hearne C, Herv V, Brune A. Comparative analysis of *Brucepastera parasyntrophica* gen. nov., sp. nov. and *Teretinema zuelzerae* gen. nov., comb. nov. (*Treponemataceae*) reveals the importance of interspecies hydrogen transfer in the energy metabolism of spirochetes. Appl Environ Microbiol. 2022;88:e00503-22. 10.1128/aem.00503-22. 35862663 10.1128/aem.00503-22 PMC9317865

[48] Buyuktimkin B, Zafar H, Saier MH. Comparative genomics of the transportome of ten *Treponema* species. Microb Pathog. 2019;132:87–99. 10.1016/j.micpath.2019.04.034. 31029716 10.1016/j.micpath.2019.04.034 PMC7085940

[49] Chow V, Nong G, Preston JF. Structure, function, and regulation of the aldouronate utilization gene cluster from *Paenibacillus* sp. strain JDR-2. J Bacteriol. 2007;189:8863–70. 10.1128/jb.01141-07. 17921311 10.1128/JB.01141-07 PMC2168633

[50] StJohn FJ, Rice JD, Preston JF. *Paenibacillus* sp. strain JDR-2 and XynA1: a novel system for methylglucuronoxylan utilization. Appl Environ Microbiol. 2006;72:1496–506. 10.1128/AEM.72.2.1496-1506.2006. 16461704 10.1128/AEM.72.2.1496-1506.2006 PMC1392964

[51] Itoh T, Ochiai A, Mikami B, Hashimoto W, Murata K. A novel glycoside hydrolase family 105: the structure of family 105 unsaturated rhamnogalacturonyl hydrolase complexed with a disaccharide in comparison with family 88 enzyme complexed with the disaccharide. J Mol Biol. 2006;360:573–85. 10.1016/j.jmb.2006.04.047. 16781735 10.1016/j.jmb.2006.04.047

[52] Richardson JS, Oresnik IJ. L-rhamnose transport is sugar kinase (RhaK) dependent in *Rhizobium leguminosarum* bv. *trifolii*. J Bacteriol. 2007;189:8437–46. 10.1128/jb.01032-07. 17890304 10.1128/JB.01032-07 PMC2168956

[53] Uehara T, Park JT. The N-acetyl-d-glucosamine kinase of *Escherichia coli* and its role in murein recycling. J Bacteriol. 2004;186:7273–9. 10.1128/jb.186.21.7273-7279.2004. 15489439 10.1128/JB.186.21.7273-7279.2004 PMC523203

[54] Salgado JFM, Hervé V, Vera MAG, Tokuda G, Brune A. Unveiling lignocellulolytic potential: a genomic exploration of bacterial lineages within the termite gut. Microbiome. 2024;12:201. 10.1186/s40168-024-01917-7. 39407345 10.1186/s40168-024-01917-7 PMC11481507

[55] Lilburn TG, Kim KS, Ostrom NE, Byzek KR, Leadbetter JR, Breznak JA. Nitrogen fixation by symbiotic and free-living spirochetes. Science. 2001;292:2495–8. 10.1126/science.1060281. 11431569 10.1126/science.1060281

[56] Skerman VBD, McGowan V, Sneath PHA. Approved lists of bacterial names. Int J Syst Evol Microbiol. 1980;30:225–420. 10.1099/00207713-30-1-225.

[57] Oren A, Arahal DR, Göker M, Moore ERB, Rossello-Mora R, Sutcliffe IC. International code of nomenclature of prokaryotes. Prokaryotic code (2022 revision). Int J Syst Evol Microbiol. 2023;73:005585. 10.1099/ijsem.0.005585. 10.1099/ijsem.0.005585 37219928

[58] Gupta RS, Mahmood S, Adeolu M. A phylogenomic and molecular signature based approach for characterization of the phylum *Spirochaetes* and its major clades: proposal for a taxonomic revision of the phylum. Front Microbiol. 2013. 10.3389/fmicb.2013.00217. 23908650 10.3389/fmicb.2013.00217 PMC3726837

[59] Radek R, Strassert JFH, Krüger J, Meuser K, Scheffrahn RH, Brune A. Phylogeny and ultrastructure of *Oxymonas jouteli*, a rostellum-free species, and *Opisthomitus longiflagellatus* sp. nov., oxymonadid flagellates from the gut of *Neotermes jouteli*. Protist. 2014;165:384–99. 10.1016/j.protis.2014.04.003. 24878512 10.1016/j.protis.2014.04.003

[60] Trager W. The cultivation of a cellulose-digesting flagellate, *Trichomonas termopsidis*, and of certain other termite protozoa. Biol Bull. 1934;66:182–90. 10.2307/1537331.

[61] Quast C, Pruesse E, Yilmaz P, Gerken J, Schweer T, Yarza P, et al. The SILVA ribosomal RNA gene database project: improved data processing and web-based tools. Nucleic Acids Res. 2013;41:D590–6. 10.1093/NAR/GKS1219. 23193283 10.1093/nar/gks1219 PMC3531112

[62] Mies US, Zheng H, Platt K, Radek R, Paczia N, Treitli SC, et al. Comparative genomics of *Elusimicrobiaceae *(phylum *Elusimicrobiota*) and description of the isolates *Elusimicrobium simillimum* sp. nov., *Elusimicrobium posterum* sp. nov., and *Parelusimicrobium proximum* gen. nov. sp. nov. Syst Appl Microbiol. 2025;48:126606. 10.1016/j.syapm.2025.126606. 40273542 10.1016/j.syapm.2025.126606

[63] Ludwig W, Strunk O, Westram R, Richter L, Meier H, Yadhukumar, et al. ARB: a software environment for sequence data. Nucleic Acids Res. 2004;32:1363–71. 10.1093/nar/gkh293. 10.1093/nar/gkh293 PMC390282 14985472

[64] Behrens S, Fuchs BM, Mueller F, Amann R. Is the in situ accessibility of the 16S rRNA of *Escherichia coli* for Cy3-labeled oligonucleotide probes predicted by a three-dimensional structure model of the 30S ribosomal subunit? Appl Environ Microbiol. 2003;69:4935–41. 10.1128/AEM.69.8.4935-4941.2003. 12902289 10.1128/AEM.69.8.4935-4941.2003 PMC169109

[65] Manz W, Amann R, Ludwig W, Wagner M, Schleifer K-H. Phylogenetic oligodeoxynucleotide probes for the major subclasses of Proteobacteria: problems and solutions. Syst Appl Microbiol. 1992;15:593–600. 10.1016/S0723-2020(11)80121-9.

[66] Weisburg WG, Barns SM, Pelletier DA, Lane DJ. 16S ribosomal DNA amplification for phylogenetic study. J Bacteriol. 1991;173:697–703. 10.1128/jb.173.2.697-703.1991. 1987160 10.1128/jb.173.2.697-703.1991 PMC207061

[67] Oberacker P, Stepper P, Bond DM, Höhn S, Focken J, Meyer V, et al. Bio-On-Magnetic-Beads (BOMB): open platform for high-throughput nucleic acid extraction and manipulation. PLoS Biol. 2019;17:e3000107. 10.1371/journal.pbio.3000107. 30629605 10.1371/journal.pbio.3000107 PMC6343928

[68] Spurr AR. A low-viscosity epoxy resin embedding medium for electron microscopy. J Ultrastruct Res. 1969;26:31–43. 10.1016/S0022-5320(69)90033-1. 4887011 10.1016/s0022-5320(69)90033-1

[69] Reynolds ES. The use of lead citrate at high pH as an electron-opaque stain in electron microscopy. J Cell Biol. 1963;17:208–12. 13986422 10.1083/jcb.17.1.208 PMC2106263

[70] Chen S, Zhou Y, Chen Y, Gu J. fastp: an ultra-fast all-in-one FASTQ preprocessor. Bioinformatics. 2018;34:i884-90. 10.1093/BIOINFORMATICS/BTY560. 30423086 10.1093/bioinformatics/bty560 PMC6129281

[71] Bankevich A, Nurk S, Antipov D, Gurevich AA, Dvorkin M, Kulikov AS, et al. SPAdes: a new genome assembly algorithm and its applications to single-cell sequencing. J Comput Biol. 2012;19:455–77. 10.1089/cmb.2012.0021. 22506599 10.1089/cmb.2012.0021 PMC3342519

[72] Dick GJ, Andersson AF, Baker BJ, Simmons SL, Thomas BC, Yelton AP, et al. Community-wide analysis of microbial genome sequence signatures. Genome Biol. 2009;10:R85. 10.1186/gb-2009-10-8-r85. 19698104 10.1186/gb-2009-10-8-r85 PMC2745766

[73] Parks DH, Imelfort M, Skennerton CT, Hugenholtz P, Tyson GW. CheckM: assessing the quality of microbial genomes recovered from isolates, single cells, and metagenomes. Genome Res. 2015;25:1043–55. 10.1101/GR.186072.114. 25977477 10.1101/gr.186072.114 PMC4484387

[74] Chklovski A, Parks DH, Woodcroft BJ, Tyson GW. CheckM2: a rapid, scalable and accurate tool for assessing microbial genome quality using machine learning. Nat Methods. 2023;20:1203–12. 10.1038/s41592-023-01940-w. 37500759 10.1038/s41592-023-01940-w

[75] Seemann T. Prokka: rapid prokaryotic genome annotation. Bioinformatics. 2014;30:2068–9. 10.1093/bioinformatics/btu153. 24642063 10.1093/bioinformatics/btu153

[76] Kanehisa M, Sato Y, Morishima K. BlastKOALA and GhostKOALA: KEGG tools for functional characterization of genome and metagenome sequences. J Mol Biol. 2016;428:726–31. 10.1016/j.jmb.2015.11.006. 26585406 10.1016/j.jmb.2015.11.006

[77] Aramaki T, Blanc-Mathieu R, Endo H, Ohkubo K, Kanehisa M, Goto S, et al. KofamKOALA: KEGG ortholog assignment based on profile HMM and adaptive score threshold. Bioinformatics. 2020;36:2251–2. 10.1093/bioinformatics/btz859. 31742321 10.1093/bioinformatics/btz859 PMC7141845

[78] Buchfink B, Reuter K, Drost H-G. Sensitive protein alignments at tree-of-life scale using DIAMOND. Nat Methods. 2021;18:366–8. 10.1038/s41592-021-01101-x. 33828273 10.1038/s41592-021-01101-x PMC8026399

[79] Zheng J, Ge Q, Yan Y, Zhang X, Huang L, Yin Y. dbCAN3: automated carbohydrate-active enzyme and substrate annotation. Nucleic Acids Res. 2023;51:W115-21. 10.1093/nar/gkad328. 37125649 10.1093/nar/gkad328 PMC10320055

[80] Emms DM, Kelly S. OrthoFinder: phylogenetic orthology inference for comparative genomics. Genome Biol. 2019;20:238. 10.1186/s13059-019-1832-y. 31727128 10.1186/s13059-019-1832-y PMC6857279

[81] Nielsen H. Predicting secretory proteins with SignalP. In: Kihara D, editor. Protein function prediction: methods and protocols. New York, NY: Springer; 2017:59–73. 10.1007/978-1-4939-7015-5_6. 10.1007/978-1-4939-7015-5_6 28451972

[82] Pruesse E, Peplies J, Glöckner FO. SINA: accurate high-throughput multiple sequence alignment of ribosomal RNA genes. Bioinformatics. 2012;28:1823–9. 10.1093/bioinformatics/bts252. 22556368 10.1093/bioinformatics/bts252 PMC3389763

[83] Wong TKF, Ly-Trong N, Ren H, Baños H, Roger AJ, Susko E, et al. IQ-TREE 3: phylogenomic inference software using complex evolutionary models. EcoEvoRxiv. 2025. 10.32942/X2P62N. https://ecoevorxiv.org/repository/view/8916/. 10.1093/molbev/msag117 PMC13191116 42085559

[84] Chaumeil PA, Mussig AJ, Hugenholtz P, Parks DH. GTDB-Tk: a toolkit to classify genomes with the Genome Taxonomy Database. Bioinformatics. 2020;36:1925–7. 10.1093/BIOINFORMATICS/BTZ848. 10.1093/bioinformatics/btz848 PMC7703759 31730192

[85] Parks DH, Chuvochina M, Chaumeil PA, Rinke C, Mussig AJ, Hugenholtz P. A complete domain-to-species taxonomy for Bacteria and Archaea. Nat Biotechnol. 2020;38:1079–86. 10.1038/s41587-020-0501-8. 32341564 10.1038/s41587-020-0501-8

[86] Wang H-C, Minh BQ, Susko E, Roger AJ. Modeling site heterogeneity with posterior mean site frequency profiles accelerates accurate phylogenomic estimation. Syst Biol. 2018;67:216–35. 10.1093/sysbio/syx068. 28950365 10.1093/sysbio/syx068

