# Supplementary material for: Metabolic capacities of large “pillotinaceous” spirochetes from termite guts and their placement among ﻿Breznakiellaceae
Source: BMC Biol. 2026 Apr 7;24:96. doi: 10.1186/s12915-026-02591-x (PMC13067680; doi:10.1186/s12915-026-02591-x)
Supplement: Supplementary file 1 — Additiopnal file 1. Supplementary figures S1-S2. Fig. S1 – Expanded 16S rRNA gene-based maximum-likelihood (ML) tree of the phylum Spirochatetota. Fig. S2 – Phylogeny of the Kalotermes termites based on cytochrome-c oxidase subunit II. [file 12915_2026_2591_MOESM1_ESM.pdf]

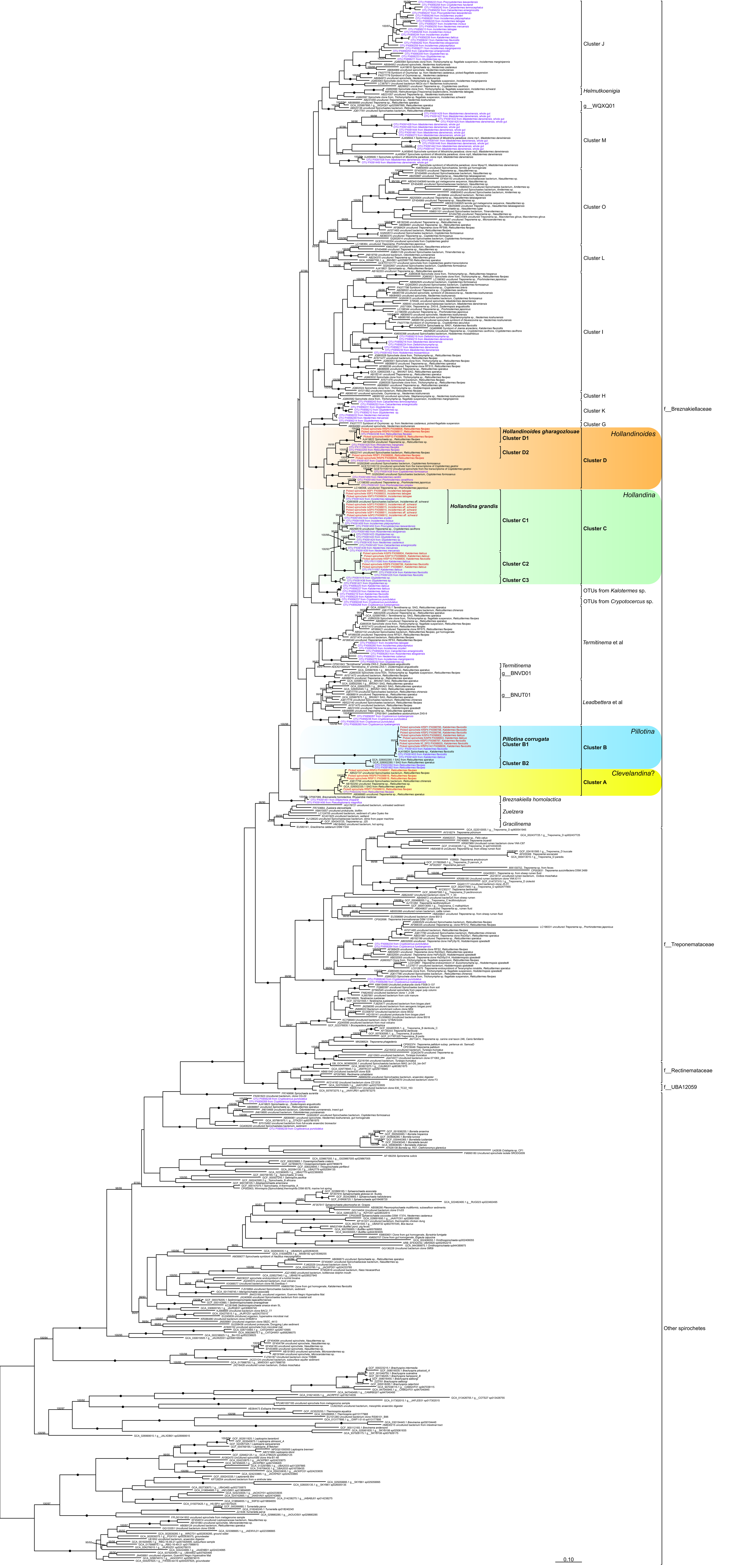

Figure S1: 16S rRNA gene-based maximum-likelihood (ML) tree of the phylum *Spirochatetota*, illustrating the relationship of the phylotypes obtained in this study to other members of the family *Breznakiellaceae*. Values at the nodes represent ML ultrafast bootstrap support values (>90%) and SH-aLRT scores (>80). Bullets represent full node support (ML: 100%; SH-aLRT: 100%). Sequences obtained from single cells are in red and sequences from amplicon libraries are in purple. The clades containing sequences from symbionts of *Mixotricha paradoxa* are highlighted in green. Scale bar, 0.1 expected substitutions per site.

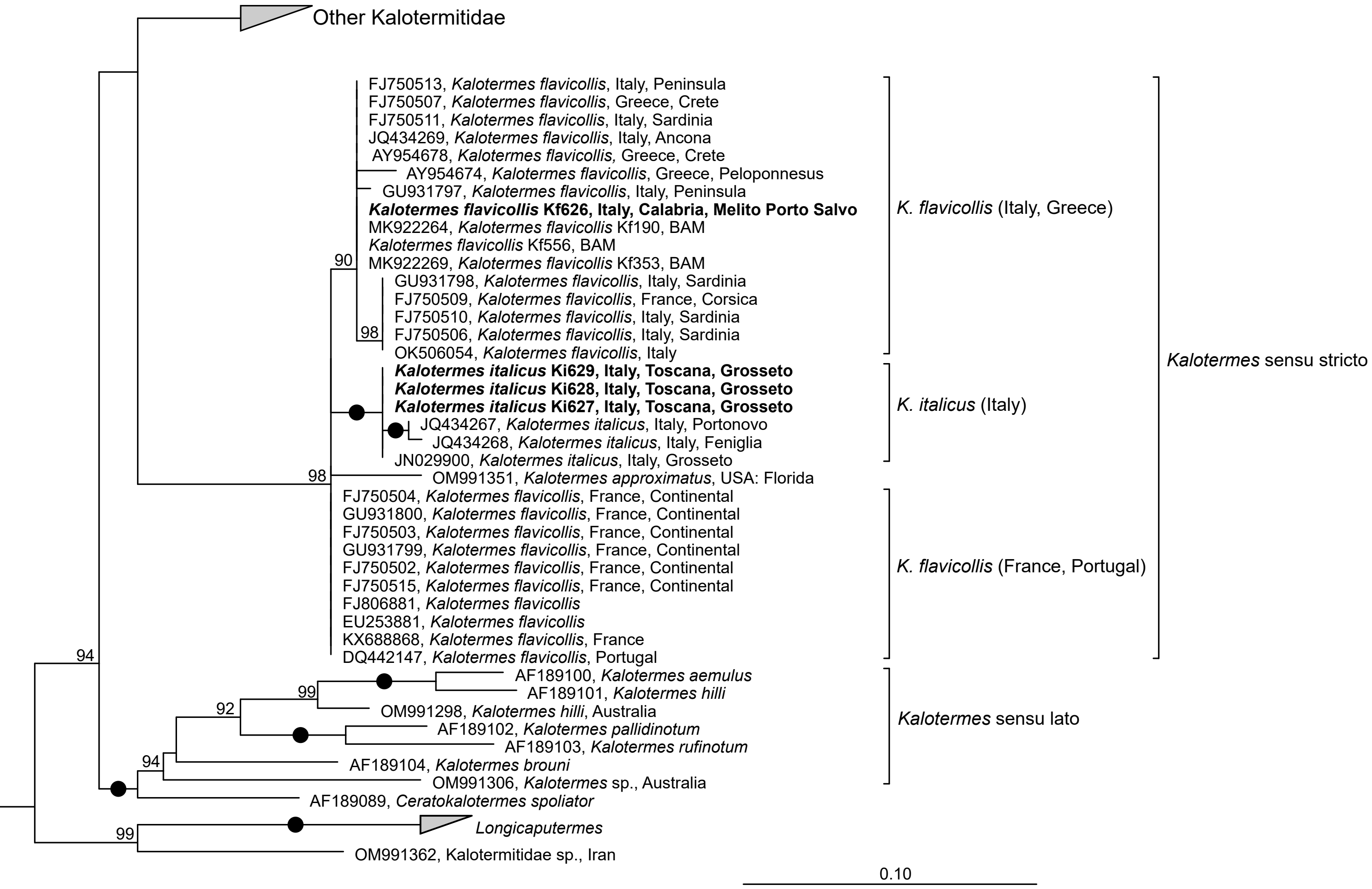

Figure S2: Phylogeny of the *Kalotermes* termites based on cytochrome-*c* oxidase subunit II (COII).The maximum-likelihood tree is based on a curated alignment of 294 amino acid positions and was inferred using IQ-Tree 2 with Model Finder Plus. Values at the nodes represent ML ultrafast bootstrap support values (>90%). Dots represent full node support (ML: 100%). Sequences derived from termites used in this study are bolded.
