# Supplementary material for: Metabolic capacities of large “pillotinaceous” spirochetes from termite guts and their placement among ﻿Breznakiellaceae
Source: BMC Biol. 2026 Apr 7;24:96. doi: 10.1186/s12915-026-02591-x (PMC13067680; doi:10.1186/s12915-026-02591-x)
Supplement: Supplementary file 3 — Additional file 3. Supplementary table S7. Table S7 – Protologues for the new species described under SeqCode. [file 12915_2026_2591_MOESM3_ESM.pdf]

Table S5. Protologues for the new species described under SeqCode.

Species name	*Pillotina corrugata*	*Hollandina grandis*	*Hollandinoides gharagozlouae*
Guiding code for nomenclature	SeqCode	SeqCode	SeqCode
Nature of the type material	Single-cell amplified genome (SAG)	Single-cell amplified genome (SAG)	Single-cell amplified genome (SAG)
Genus name	*Pillotina* (*ex* Hollande and Gharagozlou 1967) Bermudes et al. 1988	*Hollandina* (*ex* Hollande and Gharagozlou 1967) Bermudes et al. 1988	*Hollandinoides* (Hol.lan.di.no'i.des. N.L. neut. n. *Hollandina*, a genus of spirochetes; L. neut. suff. -*oides*, -like, similar; N.L. neut. n. *Hollandinoides*, a *Hollandina*-like genus.
Specific epithet	*corrugata*	*grandis*	*gharagozlouae*
Species status	sp. nov.	sp. nov.	gen. nov. sp. nov.
Descriptor	Treitli and Brune	Treitli and Brune	Treitli and Brune
Species etymology	cor.ru.ga'ta. L. fem. part. adj. *corrugata*, wrinkled, corrugated.	gran'dis. L. fem. adj. *grandis*, large, big.	gha.ra.goz.lou'i.ae. N.L. gen. n. *gharagozlouiae*, in honor of Iran Dokht Gharagozlou, an Iranian/French structural biologist
Designated type	Kf_SPG^{TS}	ItSP2^{TS}	RfSP5^{TS}
Type genome	JBQMQA000000000^{TS}	JBQLJB000000000^{TS}	JBUAPY000000000 ^{TS}
Raw data	SAMN50451124	SAMN50451380	SAMN54799087
Registry list	https://seqco.de/r:udhxf-g0	https://seqco.de/r:udhxf-g0	https://seqco.de/r:udhxf-g0
Genome status	Draft – 97.4% completeness	Draft – 95.5% completeness	Draft – 91.7% completeness
Genome size (kbp)	3056	3553	3691
GC content (mol%)	43.2	49.1	47.0
16S rRNA gene accession no.	PX098835	PX098833	PX098808
Description and diagnostic traits	The species comprises only single-cell and metagenome-assembled genomes. The species includes all bacteria with more than 95% average nucleotide identity (ANI) to the type genome.		
Isolation source	Hindgut	Hindgut	Hindgut
Host organism	*Kalotermes flavicollis* laboratory colony maintained at Federal Institute for Materials Research and Testing (BAM), Berlin, Germany	*Incisitermes tabogae* collected in the field	*Reticulitermes flavipes* laboratory colony maintained at Federal Institute for Materials Research and Testing (BAM), Berlin, Germany
Country of origin	Unknown	France: Guadeloupe	Unknown
Date of isolation	2023-10-18	2023-11-23	2025-01-13
Source of isolation			
Latitude		16.510817 N	

Longitude		61.465483 W	
Information related to the Nagoya Protocol	N/A	ABSCH-IRCC-FR-255425-1	N/A
